# In vitro selection of macrocyclic peptide inhibitors containing cyclic γ^2,4^-amino acids targeting the SARS-CoV-2 main protease

**DOI:** 10.1038/s41557-023-01205-1

**Published:** 2023-05-22

**Authors:** Takashi Miura, Tika R. Malla, C. David Owen, Anthony Tumber, Lennart Brewitz, Michael A. McDonough, Eidarus Salah, Naohiro Terasaka, Takayuki Katoh, Petra Lukacik, Claire Strain-Damerell, Halina Mikolajek, Martin A. Walsh, Akane Kawamura, Christopher J. Schofield, Hiroaki Suga

**Affiliations:** 1 Department of Chemistry, Graduate School of Science, The University of Tokyo, Tokyo, Japan; 2 Department of Chemistry and the Ineos Oxford Institute for Antimicrobial Research, Chemistry Research Laboratory, University of Oxford, Oxford, UK; 3 Diamond Light Source, Harwell Science & Innovation Campus, Didcot, UK; 4 Research Complex at Harwell, Harwell Science & Innovation Campus, Didcot, UK; 5 Chemistry – School of Natural and Environmental Sciences, Newcastle University, Newcastle upon Tyne, UK

**Keywords:** Peptides, X-ray crystallography, High-throughput screening, Translation

## Abstract

γ-Amino acids can play important roles in the biological activities of natural products; however, the ribosomal incorporation of γ-amino acids into peptides is challenging. Here we report how a selection campaign employing a non-canonical peptide library containing cyclic γ^2,4^-amino acids resulted in the discovery of very potent inhibitors of the SARS-CoV-2 main protease (M^pro^). Two kinds of cyclic γ^2,4^-amino acids, *cis*-3-aminocyclobutane carboxylic acid (γ^1^) and (1*R*,3*S*)-3-aminocyclopentane carboxylic acid (γ^2^), were ribosomally introduced into a library of thioether-macrocyclic peptides. One resultant potent M^pro^ inhibitor (half-maximal inhibitory concentration = 50 nM), GM4, comprising 13 residues with γ^1^ at the fourth position, manifests a 5.2 nM dissociation constant. An M^pro^:GM4 complex crystal structure reveals the intact inhibitor spans the substrate binding cleft. The γ^1^ interacts with the S1′ catalytic subsite and contributes to a 12-fold increase in proteolytic stability compared to its alanine-substituted variant. Knowledge of interactions between GM4 and M^pro^ enabled production of a variant with a 5-fold increase in potency.

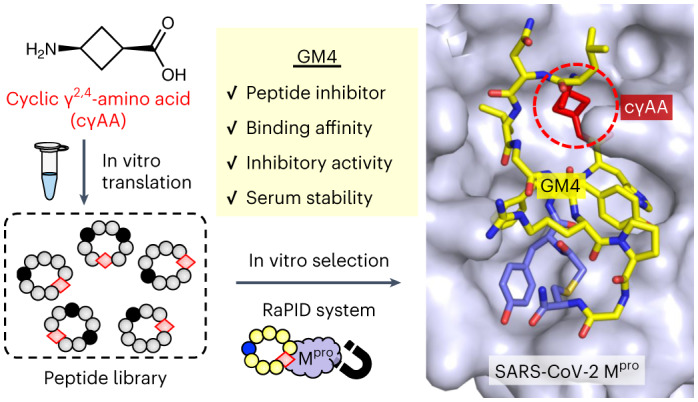

## Abstract (web summary)

In vitro screening of a ribosomally synthesized macrocyclic peptide library containing cyclic γ^2,4^-amino acids (cγAA) afforded the discovery of potent inhibitors of the SARS-CoV-2 main protease (M^pro^). A co-crystal structure revealed the contribution of this cγAA to M^pro^ binding and the proteolytic stability of these macrocycles.

## Main

γ-Amino acid residues can induce unique peptide conformations, such as C_14_-helix, C_12/10_-helix and C_12_-turn secondary structures^1–10^. The γ-amino acids present in natural products play important roles in their biological activities and enable improved proteolytic stability and cell permeability^11,12^. Pepstatin, isolated from *Actinomyces*, has two γ-amino acid residues, which contribute to its protease inhibitory activity by enhancing binding to the active sites^13–15^. Didemnin, an antiviral/antitumor compound, has a γ-amino acid and a macrocyclic scaffold^16^. Given their remarkable bioactivities and conformational rigidity, macrocyclic peptides containing γ-amino acids are an attractive option for incorporation into novel therapeutic peptides.

Over the past two decades, the ribosomal synthesis and selection-based screening of peptides containing nonproteinogenic amino acids, including *N*-methyl-l-α-amino, d-amino and β-amino acids, have been accomplished^17–26^. However, the ribosomal incorporation of γ-amino acids into peptides has been challenging for the following three reasons: (1) efficient self-deacylation of γ-aminoacyl-tRNA via stereoelectronically favoured 5-oxo-trig cyclization, (2) slow accommodation of γ-aminoacyl-tRNAs into the ribosome A site and (3) slow peptidyl transfer of the P-site peptidyl-tRNA onto the γ-amino group of the A-site γ-aminoacyl-tRNA due to poor compatibility with the peptidyl transferase centre of the ribosome^27,28^. We recently reported that the self-deacylation issue can be circumvented by using structurally constrained cyclic γ^2,4^-amino acids (cγAAs)^29^. The cγAAs were charged on an engineered tRNA, referred to as tRNA^Pro1E2^ (Supplementary Fig. 1), the T-stem and D-arm of which were optimized for efficient binding to elongation factor Tu (EF-Tu) and EF-P, thereby accelerating accommodation and peptidyl transfer, respectively^30–32^. Combination of the flexizyme-assisted^33,34^ synthesis of cγAA–tRNA^Pro1E2^ and the reconstituted *Escherichia coli* translation system, referred to as the Flexible In vitro Translation (FIT) system^23^, that contains EF-Tu and EF-P with their adjusted concentrations enabled the incorporation of cγAAs into nascent peptide chains^29,35,36^.

**Fig. 1:**
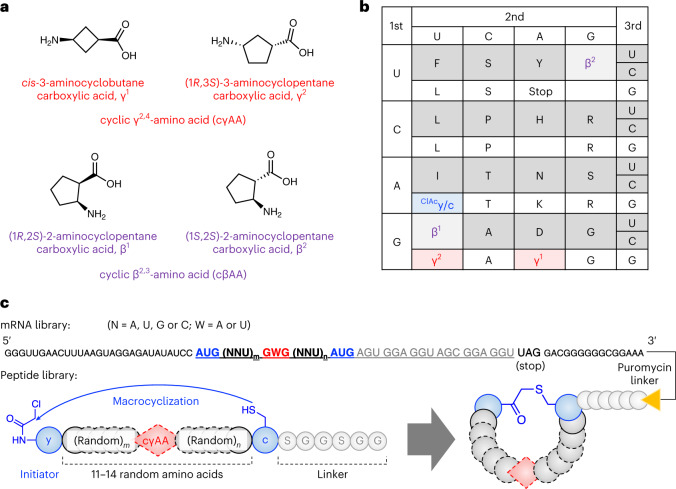
Ribosomal incorporation of cγAAs into a macrocyclic peptide library. **a**, Structures of the cγAAs and cβAAs used in this study. **b**, The reprogrammed codon table that contains ^ClAc^y at the initiator AUG codon, and c, γ^1^, γ^2^, β^1^ and β^2^ at the elongator AUG, GAG, GUG, GUU and UGU codons, respectively. NNA codons are omitted because they were not used in the mRNA library. **c**, Construction of the random mRNA library and the corresponding peptide library. Peptides spontaneously macrocyclized between ^ClAc^y and c via a thioether bond. The mRNA and peptide were covalently linked via a puromycin linker. Source data

Here we report the ribosomal synthesis of a macrocyclic peptide library containing cγAAs and its application to an in vitro display, referred to as the Random nonstandard Peptides Integrated Discovery (RaPID) system^23^. The library contains two types of cγAAs, *cis*-3-aminocyclobutane carboxylic acid (γ^1^) and (1*R*,3*S*)-3-aminocyclopentane carboxylic acid (γ^2^) (Fig. 1a). Owing to the constrained rigid conformations of these cγAAs, they can be exquisite building blocks for preorganization of peptide secondary structures. Therefore, screening of macrocyclic peptide libraries containing cγAAs can lead to molecules with potent binding affinities and peptidase resistance. As a biomedicinally important test target protein, we chose M^pro^, which plays an essential role in processing the polyproteins translated from the viral RNA^37^. Peptidomimetic inhibitors of M^pro^ have been shown to be therapeutically valuable for the treatment of coronavirus disease 2019 (COVID-19), with one approved for clinical use (nirmatrelvir, PF-07321332)^38^. Importantly, however, most of these inhibitors contain a reactive warhead (a nitrile in the case of nirmatrelvir) that reacts to form a covalent bond with a Cys residue in the M^pro^ active site. We took up the challenge of discovering potent ‘non-covalent’ active site binding M^pro^ inhibitors by applying the RaPID system with cγAA-containing macrocyclic peptide libraries.

## Results

### Selection of macrocyclic peptide binders to SARS-CoV-2 M^pro^

To construct a cγAA-containing macrocyclic peptide library, γ^1^ and γ^2^ (Fig. 1a) were assigned to GAG and GUG codons using tRNA^Pro1E2^_CUC_ and tRNA^Pro1E2^_CAC_, respectively (Fig. 1b), employing the FIT system. Two cyclic β^2,3^-amino acids (cβAAs), (1*R*,2*S*)-2-aminocyclopentane carboxylic acid (β^1^) and (1*S*,2*S*)-2-aminocyclopentane carboxylic acid (β^2^), were introduced at the GUU and UGU codons using tRNA^Pro1E2^_GAC_ and tRNA^GluE2^_GCA_, respectively, because we considered that the incorporation not only of cγAA, but also of cβAA, would give more diverse folding possibilities^25^. For the macrocyclization of the library via thioether bonds, we introduced *N*-chloroacetyl-d-tyrosine (^ClAc^y) and d-cysteine (c) at the initiator AUG codon using tRNA^fMet^_CAU_ and elongator AUG codon using tRNA^Pro1E2^_CAU_, respectively. The thioether bond spontaneously forms between the *N*-terminal chloroacetyl group on ^ClAc^y and the thiol group of the c residue (Fig. 1c). These amino acids were precharged onto the respective tRNAs using flexizymes. The peptide library comprised a repeat of 11−14 random residues encoded by GWG and NNU codons (W = A or U; N = A, U, G or C) flanked by the cyclizing ^ClAc^y and c residues. Since multiple cγAA incorporations could not be efficiently achieved, as reported in our previous study^29^, the library was designed to have only a single γ^1^ or γ^2^ appear at the GWG codon. The two cβAAs and the thirteen proteinogenic l-α-amino acids (A, D, F, G, H, I, L, N, P, R, S, T and Y) were assigned at NNU codons. The C-terminal SGGSGG sequence following the c residue was a linker peptide connected to the 3′ end of mRNA via a puromycin linker. To demonstrate the fidelity of translation, model macrocyclic peptides containing any of cγAA and cβAA (γ^1^, γ^2^, β^1^ and β^2^ assigned at GAG, GUG, GUU and UGU codons, respectively) were synthesized, and their identities were confirmed by matrix-assisted laser desorption/ionization coupled with time of flight mass spectrometry (MALDI-TOF MS; Supplementary Fig. 2).

**Extended Data Fig. 1:**
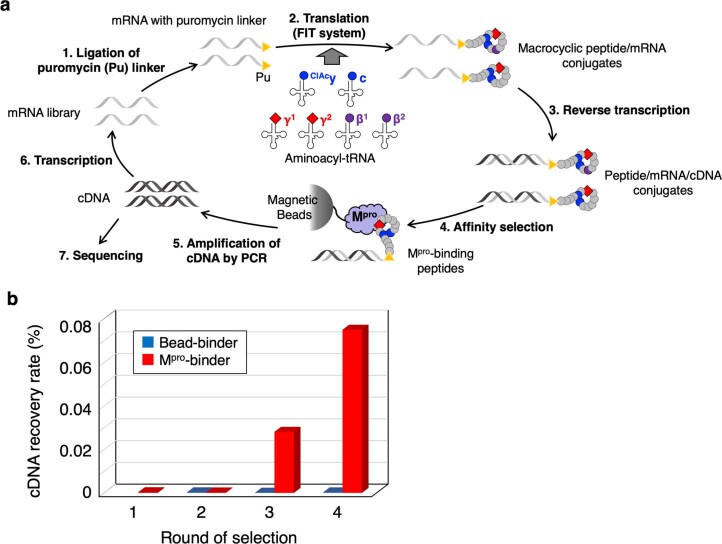
RaPID selection against M^pro^ using a macrocyclic peptide library containing cγAA. **a**, Schematic depiction of RaPID display. 1) Puromycin linker ligation to the 3′-end of the mRNA library. 2) Translation of peptides using the reprogrammed genetic code, followed by spontaneous macrocyclization of peptide via a thioether bond. 3) Reverse transcription of mRNA into cDNA. 4) Binding selection of peptides against M^pro^ immobilized on magnetic beads. 5) Recovery of the bound fraction and amplification of cDNA by PCR. 6) Transcription of cDNA library into mRNA library. 7) Deep sequencing analysis of cDNA library. **b**, Recovery rate of cDNA after the binding selection at each round. Red and blue bars indicate the recovery rate of M^pro^ binders and magnetic beads binders, respectively. Bead-binder selection was not performed in the first round.

The macrocyclic peptide library was then applied to the RaPID selection against recombinant SARS-CoV-2 M^pro^. Translation of the random mRNA library into the peptide library, conjugation of the peptide with the parent mRNA via a puromycin linker and reverse transcription of the mRNA into the cDNA yielded peptide/mRNA/cDNA conjugates (Extended Data Fig. 1a). The library was first subjected to naked magnetic bead treatment to remove bead binders, and then applied to M^pro^-immobilized magnetic beads to recover M^pro^ binders. The cDNA moiety of the recovered peptide/mRNA/cDNA conjugate was amplified by the polymerase chain reaction (PCR) and transcribed into the mRNA library for the next selection round (details in Methods). By repeating this affinity selection, the recovery rate of the M^pro^ binders was greatly increased at the third round of selection and, even more obviously, in the fourth round, while the recovery of the bead binders did not increase (Extended Data Fig. 1b). Deep sequencing of the cDNA library at the fourth round revealed that the library was enriched with several families of peptides bearing cγAA (Supplementary Table 1 shows the top 100 sequences). Among these, seven macrocyclic peptides containing cγAA were selected for further analysis of their binding affinities, inhibitory activity and proteolytic stability (Table 1, GM1−GM7). GM1, GM2, GM4 and GM6 contain a γ^1^ residue in their sequences, while GM3, GM5 and GM7 contain γ^2^. Note that the cβAA-containing peptides were not selected because they were not included in the major peptide families with high read numbers. GM1−GM7 were chemically synthesized on a large scale using the standard solid-phase method without the C-terminal SGGSGG linker, and their purities and identities were confirmed by ultra-performance liquid chromatography (UPLC) and MALDI-TOF MS, respectively (Supplementary Figs. 3 and 4).

**Table 1:**
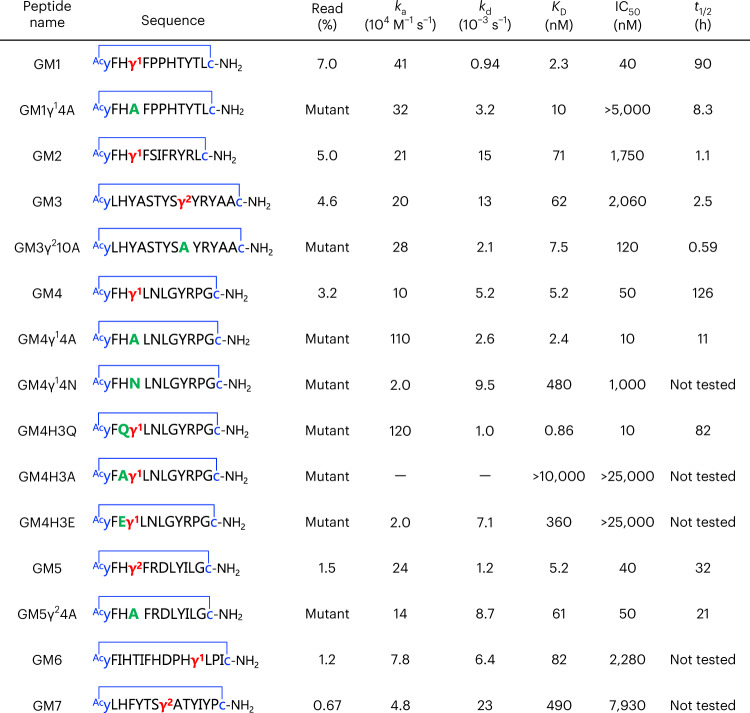
Binding affinities, inhibitory activities and serum stabilities of the macrocyclic peptides

**Fig. 2:**
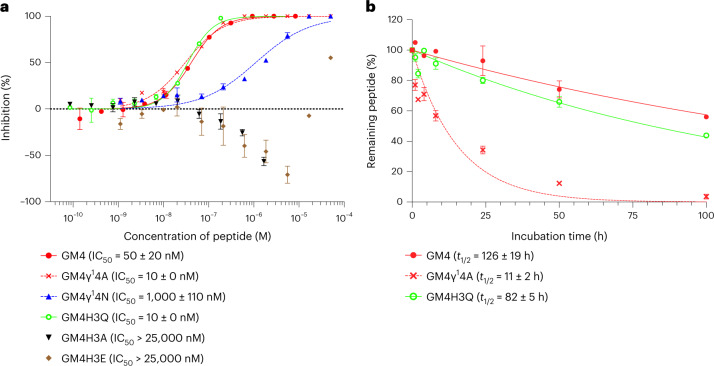
M^pro^ inhibitory activity and serum stability of GM4 and its mutants. **a**, Dose response analysis of peptides against M^pro^. The M^pro^ inhibitory activities of peptides were investigated by solid-phase extraction purification coupled to MS analysis using a RapidFire 365 high-throughput sampling robot (Agilent) connected to an iFunnel Agilent 6550 accurate mass quadrupole TOF mass spectrometer^39^. IC_50_ values were determined by the mean of three or five independent replicates each performed in technical duplicate. Data are presented as mean values ± standard deviation, s.d. (*n* = 5 for GM4, GM4γ^1^4A and GM4H3Q; *n* = 3 for GM4γ^1^4N, GM4H3A and GM4H3E). Extended Data Fig. 2 shows other peptides. **b**, Serum stability assay of macrocyclic peptides. GM4, GM4γ^1^4A and GM4H3Q were co-incubated with an internal standard peptide in human serum (37 °C). At each time point, the relative intensity of each peptide to the standard peptide was estimated by LC/MS. The relative intensity at 0 h was defined as 100%. Half-lives (*t*_1/2_) were determined by analysing the mean of three technical replicates of each sample by nonlinear regression using GraphPad Prism 9. Data are presented as mean values ± s.d. (*n* = 3). Extended Data Fig. 3 shows results for other peptides.

**Extended Data Fig. 2:**
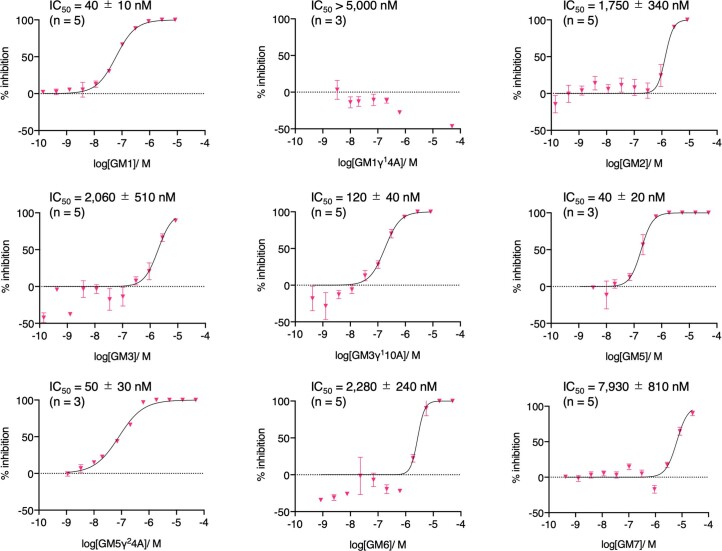
Representative dose response analyses of inhibition of M^pro^ by macrocycles. Conditions: 75 nM M^pro^, 4 µM substrate peptide, macrocyclic peptides in 20 mM HEPES, pH 7.5, 50 mM NaCl. See Methods for details. Table 1 gives the recorded IC_50_ values as the mean of 3 or 5 independent replicates each performed in technical duplicate. Data are presented as mean values ± SD. Number of replicates is indicated above the graph. See Fig. 2 for GM4 and its variants.

**Extended Data Fig. 3:**
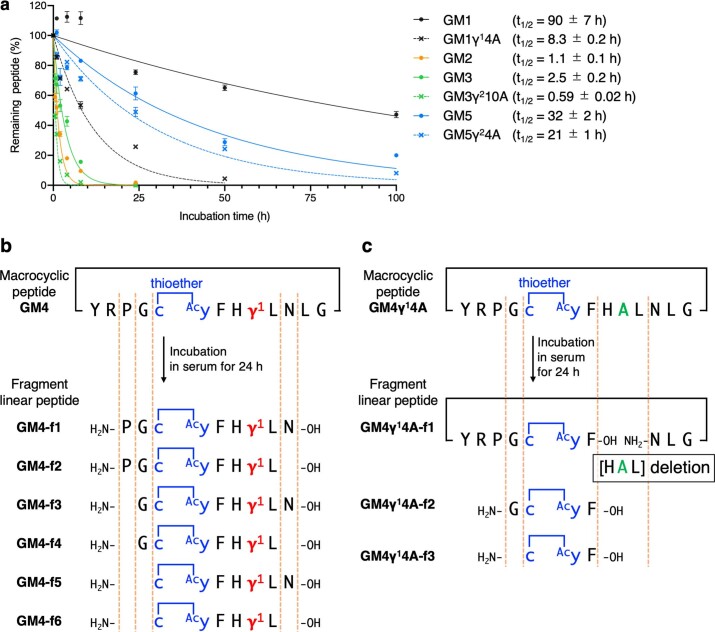
Serum stability assay of macrocyclic peptides. **a**, Each of GM1, GM1γ^1^4A, GM2, GM3, GM3γ^2^10A, GM5, and GM5γ^2^4A were co-incubated with an internal standard peptide in human serum at 37 °C. At each time point (0, 0.5, 1, 2, 4, 8, 24, 50, and 100 h), the relative intensity of each peptide with respect to the standard peptide was estimated by LC/MS. The relative intensity at 0 h was defined as 100%. Half-lives (t_1/2_) were determined by analyzing the mean of 3 technical replicates of each sample by non-linear regression using GraphPad Prism 9. Data are presented as mean values ± SD (n = 3). **b**,**c**, Fragments of GM4 (GM4-f1−6) and GM4γ^1^4A (GM4γ^1^4A-f1−3), respectively, after 24 h incubation in human serum as analyzed by LC/MS.

The thioether-macrocyclic peptides selected by the RaPID system, GM1−GM7, and their mutants are shown. The thioether bond is shown as a blue line where the sulfide atom is omitted. The γ^1^ and γ^2^ residues are in red. The table shows sequences, read (%) at the fourth round, kinetic association (*k*_a_), kinetic dissociation (*k*_d_), *K*_D_, IC_50_ and half-life in human serum (*t*_1/2_). Supplementary Fig. 5 shows the sensorgrams of SPR analysis; Fig. 2a and Extended Data Fig. 2 show the results of the M^pro^ inhibition assay; Fig. 2b and Extended Data Fig. 3 show the results of serum stability assays. −, the kinetic values could not be accurately determined due to low affinity.

### Biological activities and stabilities of peptide inhibitors

We first evaluated the binding affinity of GM1−GM7 to M^pro^ by surface plasmon resonance (SPR). All the peptides exhibited low-to-moderate nanomolar kinetic equilibrium (*K*_D_) values (Table 1 and Supplementary Fig. 5). Notably, GM1 and GM4, which contain γ^1^, and GM5, which contains γ^2^, have a conserved four residue motif, yFHγ^X^ (γ^X^ = γ^1^ or γ^2^), at their N termini and showed potent affinities, with *K*_D_ values of 2.3, 5.2 and 5.2 nM, respectively; GM2 has the same motif, but has a higher *K*_D_ (71 nM). GM6 and GM7, which had relatively low read frequencies, exhibited weak binding compared to GM1−GM5, indicating selection for tight binding peptides. To evaluate contributions of the cγAA residues to potency, we synthesized peptides where the cγAA was substituted with alanine (Table 1, GM1γ^1^4A, GM3γ^2^10A, GM4γ^1^4A and GM5γ^2^4A). In addition to GM1, GM4 and GM5, which share the conserved yFHγ^X^ motif, GM3 was selected for alanine substitution because of its high read percentage among peptides without the yFHγ^X^ motif. SPR measurements of the alanine mutants revealed that the *K*_D_ values of GM1γ^1^4A, GM4γ^1^4A and GM5γ^2^4A were comparable or one order higher than those of the original peptides, indicating that the cγAA residue in the conserved yFHγ^X^ motif is important for binding. To our surprise, GM3γ^2^10A exhibited an eightfold stronger binding affinity (*K*_D_ = 7.5 nM), revealing that the contribution of cγAA to the binding affinity is sequence context dependent.

We next evaluated the inhibitory activity of GM1−GM7 against the hydrolytic activity of SARS-CoV-2 M^pro^ using a reported MS-based method^39^. GM1−GM7 exhibited inhibition of M^pro^; in particular, the yFHγ^X^ motif containing GM1, GM4 and GM5, which manifest single-digit nanomolar *K*_D_ values, showed particularly potent inhibition, with half-maximal inhibitory concentration (IC_50_) values of 40, 50 and 40 nM, respectively (Table 1, Fig. 2a and Extended Data Fig. 2). The peptides with weaker binding affinity, that is, GM2, GM3, GM6 and GM7, had IC_50_ values of 1,750–7,930 nM, implying correlation between IC_50_ and *K*_D_ values. The inhibitory activities of the alanine mutants were then determined. Notably, despite GM1γ^1^4A having only a fourfold weaker binding affinity compared with GM1, inhibition was ablated, revealing the importance of the cγAA. By contrast, GM3γ^2^10A, GM4γ^1^4A and GM5γ^2^4A retained inhibition activity, showing the context-dependent effects of the cγAA (IC_50_ = 120, 10 and 50 nM, respectively; Table 1, Fig. 2a and Extended Data Fig. 2).

We evaluated the half-life of potent cγAA-containing peptides and their mutants in human serum, because in vivo stability is a critical factor in the development of therapeutic peptides. Each peptide and an uncleavable internal standard peptide were co-incubated in human serum at 37 °C, and the relative amount of the remaining sample peptide was estimated by liquid chromatography/MS (LC/MS). The potent inhibitors GM1 and GM4, containing γ^1^, and GM5, containing γ^2^, exhibit high peptidase resistance with half-lives (*t*_1/2_) of 90 h, 126 h and 32 h, respectively (Table 1, Fig. 2b and Extended Data Fig. 3a). Importantly, by contrast, their alanine mutants show substantially shorter half-lives (*t*_1/2_ = 8.3 h, 11 h and 21 h for GM1γ^1^4A, GM4γ^1^4A and GM5γ^2^4A, respectively; Table 1). The enhancement of serum resistance observed for GM4, for instance, was 12-fold. Notably, despite the substitution of γ^1^ to an Ala residue not diminishing inhibition relative to GM4, the enhancement in serum stability by the γ^1^ residue is substantial. Moreover, the less potent inhibitors GM2 and GM3 have more than nearly three orders of magnitude shorter half-lives (1.1 h and 2.5 h, respectively) compared to GM4, suggesting that the higher (binding and inhibitory) activity of GM4 is reflected in its higher serum stability, possibly due to its more conformationally constrained fold.

To identify peptidase cleavage sites in serum, the products of GM4 and GM4γ^1^4A after 24 h incubation were analysed by LC/MS. In the case of GM4, six fragments (GM4-f1−GM4-f6, Extended Data Fig. 3b) containing the c-(thioether)-^Ac^yFHγ^1^L motif were detected, but no fragmentation of the motif itself was apparent. With GM4γ^1^4A, three shorter fragments containing c-(thioether)-^Ac^yF were detected (GM4γ^1^4A-f1−GM4γ^1^4A-f3; Extended Data Fig. 3c), indicating cleavage between F and H of the motif. These results show that the presence of non-canonical residues (one cγAA (γ^1^), two d-amino acids and a thioether bond) can enable high peptidase resistance by preventing the proteolysis of flanking residues, leading to a remarkable improvement in serum stability.

### X-ray crystallographic analysis of the GM4:M^pro^ complex

**Fig. 3:**
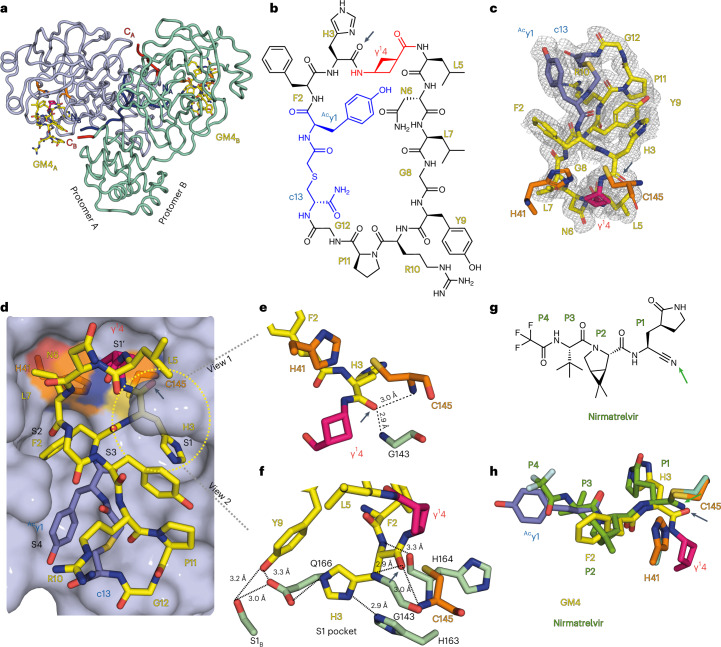
Crystallographic studies reveal the binding mode of the GM4 macrocyclic peptide at the M^pro^ active site. **a**, GM4 binds in the substrate binding cleft of both protomers A and B in the M^pro^ dimer. **b**, Structure of GM4; the H3_GM4_ carbonyl O is indicated with an arrow. **c**, Polder omit map of GM4 contoured at a level of ±1.5 standard deviation (σ). **d**, View of GM4 at the active site. The H41 and C145 catalytic dyad is in orange. γ^1^4_GM4_ (magenta) occupies the S1′ pocket; the side chains of H3_GM4_, F2_GM4_ and ^Ac^y1_GM4_ occupy the S1, S2 and S4 pockets, respectively. **e**,**f**, Close-ups of the S1 pocket, showing the H3_GM4_ backbone carbonyl in the oxyanion hole (C145, G143 backbone amides). The backbone NH of H3_GM4_ is positioned to interact with the H164 backbone CO. The H3_GM4_ imidazole is positioned to hydrogen bond with the H163 side chain and to interact with the E166 side chain, which interacts with S1 of protomer B, as observed in apo-M^pro^ structures. Y9_GM4_ is 3.1 Å and 3.3 Å from the E166 and S1_B_ side chains, respectively. **g**, Nirmatrelvir with the residues occupying the S1−S4 subsites labelled P1−P4 (the reactive nitrile is indicated with an arrow). **h**, Superimposition of views from crystal structures of M^pro^ with GM4 and nirmatrelvir (Protein Data Bank 7VH8 (ref. ^60^)). GM4 non-covalently interacts at the active site, while the nitrile of nirmatrelvir reacts with C145. Note the similar locations of the nirmatrelvir nitrile-derived N and the H3_GM4_ carbonyl O.

To gain structural insights into how GM4, including its γ^1^ residue, interacts with M^pro^, we obtained an X-ray crystal structure of their complex to 1.7 Å resolution (Fig. 3a and Supplementary Table 2). The structure reveals GM4 bound with high occupancy and in the same manner at the active site regions of both monomers in the M^pro^ dimer. Excellent electron density was observed for all residues in the GM4 macrocycle (Fig. 3c). The overall conformations of M^pro^ in the GM4 and nirmatrelvir^38^ (used for comparison) complex structures are very similar (backbone root mean square deviation, 0.34).

**Extended Data Fig. 4:**
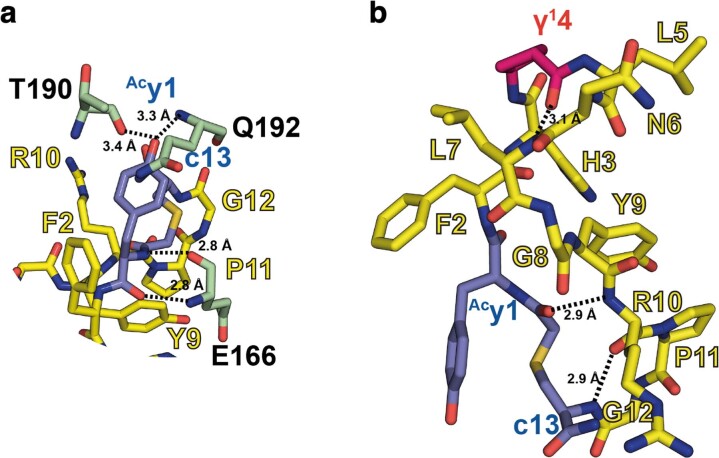
Views from crystallographic studies. **a**, Interaction of the y1_GM4_ residue at the M^pro^ active site. The phenolic sidechain of y1_GM4_ forms polar interactions with T190 and Q192. The backbone NH of y1_GM4_ is positioned to form H-bonds with the mainchain carbonyl and NH groups of E166, as is typical of P3 residues in peptidomimetic M^pro^ inhibitors (Ref. ^40^). **b**, Intramolecular hydrogen bonds in GM4; H-bonds are formed between γ^1^4_GM4_ and L7_GM4_, ^Ac^y1_GM4_ and R10 _GM4_, and P11_GM4_ and c13_GM4_. H-bonds are indicated with dashed black lines; associated distances are in Ångström.

The four residues in the yFHγ^1^ motif of GM4 occupy the S4, S2, S1 and S1′ substrate binding subsites, respectively^40^ (Fig. 3d). H3_GM4_ is accommodated in the S1 subsite, forming hydrogen bonds with M^pro^ C145, H164 and H163 (GM4 residues are identified by a subscript; Fig. 3e,f). The F2_GM4_ residue binds in the S2 subsite, making hydrophobic interactions with M49, M165 and H41. The y1_GM4_ side chain forms hydrogen bonds with T190 and Q192 in the S4 subsite (Extended Data Fig. 4a).

The c13_GM4_–thioether link, which enables macrocyclization, and the adjacent G12_GM4_ enable a turn connected to P11_GM4_, R10_GM4_ and Y9_GM4_; none of these residues are positioned to make direct contacts with M^pro^ in the structure, though these cannot be ruled out in solution. The backbone of GM4 forms three intramolecular hydrogen bonds, that is between γ^1^4_GM4_ and L7_GM4_, ^Ac^y1_GM4_ and R10_GM4_, and P11_GM4_ and c13_GM4_ (Extended Data Fig. 4b). The side chains of P11_GM4_ and Y9_GM4_ are arranged such that they ‘stack’ with each other, an arrangement positioning the phenolic side chain of Y9_GM4_ adjacent to the imidazole ring of H3_GM4_, appearing to lock it into the S1 pocket; the phenolic side chain of Y9_GM4_ also forms interactions with Ser1_,_ from the other protomer of the M^pro^ dimer (Fig. 3f).

G8_GM4,_ L7_GM4,_ N6_GM4_ and L5_GM4_ form a loop leading to γ^1^4, which is linked to H3_GM4._ γ^1^4_GM4_ is positioned to make hydrophobic contact with T24 and T25; the side chains of L5_GM4_ and N6_GM4_ are positioned close to the surface of M^pro^, with L7_GM4_ projecting towards H41, C44 and T45. The observation of the unnatural γ^1^4_GM4_ residue, the cyclobutane ring of which was refined in a near planar conformation, in the P1′ position is striking and implies it contributes to the inhibition or lack of efficient GM4 hydrolysis.

At the active site region, the sulfur of the nucleophilic C145 is positioned close (3.4 Å) to the imidazole ε-nitrogen of H41, which acts as a general base/acid in M^pro^ catalysis, and to the carbonyl carbon (4.4 Å) of the amide linking H3_GM4_ and γ^1^4_GM4_; this carbonyl carbon is positioned in a similar manner to the imine (anion) derived by reaction of the nirmatrelvir nitrile with C145 (Fig. 3g,h).

Like the binding modes of substrates (predicted) and inhibitors (as observed by crystallography), the carbonyl of the amide linking H3_GM4_ and γ^1^4_GM4_ is positioned in the oxyanion hole, making hydrogen bonds with the C145 NH (2.9 Å) and with the G143 NH (2.9 Å). The ~100° angle between the C145 sulfur and the carbon and oxygen of the amide linking H3 and γ^1^4 is close to that predicted for nucleophilic attack onto an amide carbonyl as observed in studies on serine proteases^41^. Despite this apparently catalytically productive arrangement, analysis of the electron density map implies a lack of substantial covalent reaction, consistent with solution studies showing a lack of efficient GM4 hydrolysis by M^pro^. Lack of reaction appears to be a consequence of multiple interactions made between the peptide macrocycle and M^pro^, likely involving P11_GM4_, Y9_GM4_ and H3_GM4_, resulting in tight binding in a catalytically non-productive manner.

SARS-CoV-2 M^pro^ cleaves after a conserved glutamine residue (P1) in substrates with the following sequence motif: (P2, L/F/V/M (hydrophobic))(P1, Q)↓(P1′, S/A/G/N (small)), where ↓ represents the cleavage site^40,42^. Interestingly, rather than a glutamine (Q), GM4 has a histidine (H3) at the analogous ‘P1’ position. To investigate the contribution of the H3/P1 residue for activity, we evaluated peptides bearing Q, A or E residues at the analogous position. Among these, GM4H3Q exhibited a sixfold stronger binding affinity (*K*_D_ = 0.86 nM) and fivefold more potent inhibitory activity (IC_50_ = 10 nM; Table 1, Fig. 2 and Supplementary Fig. 5). By contrast, GM4H3A manifested no inhibition, and inhibition of GM4H3E was observed only with the highest tested concentration (25 µM). These results are consistent with the known substrate selectivity of M^pro^ at the P1 position^40^.

In the case of the P1′ residue, M^pro^ prefers substrates with small residues, such as G, A and S, although N is also tolerated^40^. Strikingly, GM4 has γ^1^4 at the P1′ position, likely reflecting the compact size of the γ^1^ four-membered-ring main-chain residue. In support of this, GM4γ^1^4A (γ^1^ → A) exhibited a comparable inhibitory activity to that of GM4 (IC_50_ of GM4γ^1^4A = 10 nM), and GM4γ^1^4N was 20-fold less active (IC_50_ = 1,000 nM). Despite the almost equivalent M^pro^ inhibition activities of GM4 and GM4γ^1^4A, the serum stability of GM4 is 12-fold higher (*t*_1/2_ = 126 h) than that of GM4γ^1^4A (Fig. 2b). Furthermore, the most potent mutant GM4H3Q bearing γ^1^4 at P1 also exhibited a comparable serum stability (*t*_1/2_ = 82 h). These results show how the introduction of a nonstandard amino acid at the P1′ position can prolong the serum stability to non-targeted protease-catalysed hydrolysis while maintaining potency against the targeted protease (M^pro^ in our case).

## Discussion

**Extended Data Fig. 5:**
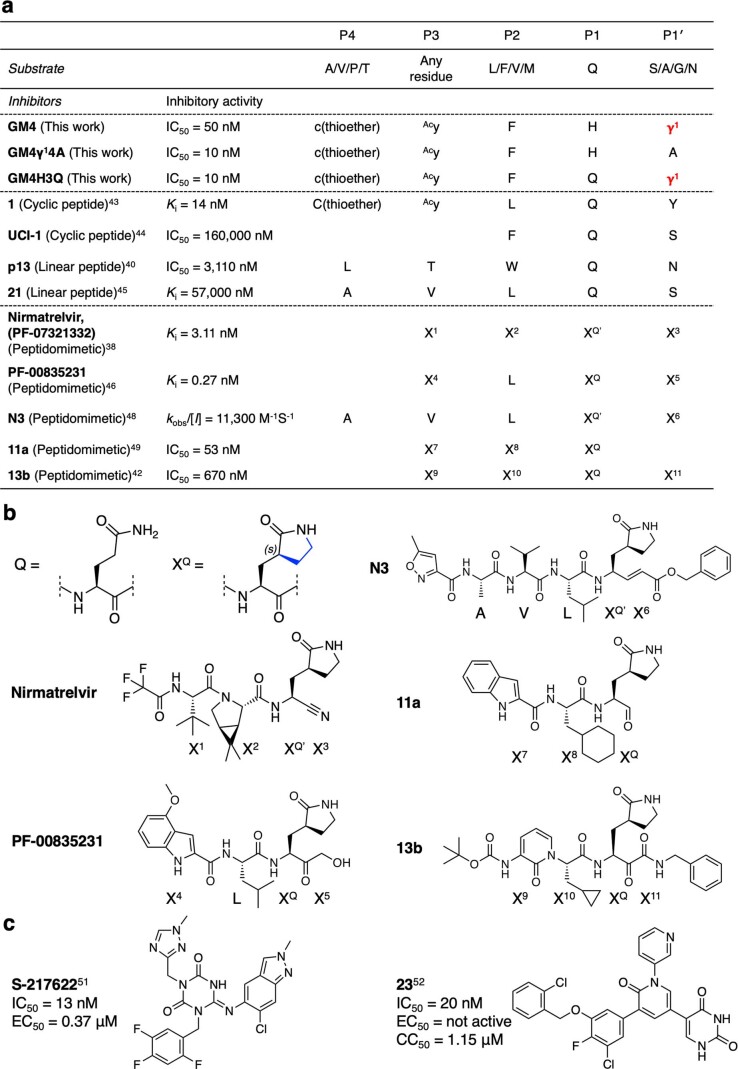
Comparison of GM4 and its variants with M^pro^ substrates and reported M^pro^ inhibitors. **a**, Amino acid sequences around the cleavage site of the M^pro^ substrate and inhibitors. Superscript numbers indicate references. **b**, Structures of glutamine analog X^Q^ and peptidomimetics. **c**, Structures and activities of non-covalent small molecule inhibitors of M^pro^.

Our results reveal GM4 and GM4H3Q as highly potent cyclic peptides targeting M^pro^. Other peptides and peptidomimetic inhibitors of SARS-CoV-2 M^pro^ have been reported with varying potencies (Extended Data Fig. 5). Johansen-Leete et al. recently used the RaPID system to screen thioether-macrocyclic peptides containing standard proteinogenic amino acids in the random region between the cyclizing ^ClAc^y and the downstream l-Cys (ref. ^43^). A macrocyclic peptide, with yLQY residues at the P3−P1′ equivalent positions, exhibiting an inhibition constant (*K*_i_) value of 14 nM against M^pro^ was identified. The P1 equivalent Q and P1′ equivalent Y residues are on either side of the scissile amide in this peptide, resulting in slow cleavage by M^pro^.

In the case of the M^pro^:GM4 complex crystal structure, well-defined electron density was observed for the complete intact peptide in the active site pocket despite the carbonyl of the amide linking H3_GM4_ and γ^1^4_GM4_ being positioned in the oxyanion hole. This observation both highlights the advantage of introducing a cγAA into the library and implies that GM4 and GM4H3Q in part resist hydrolytic cleavage due to the presence of γ^1^ at the P1′ position. Other groups have also reported a substrate-derived cyclic peptide inhibitor of M^pro^ (UCI-1 (ref. ^44^)) and linear peptide inhibitors of M^pro^ (p13 (ref. ^40^) and compound 21 (ref. ^45^)), but the potency is limited to the micromolar range. Peptidomimetic inhibitors, including nirmatrelvir (PF-07321332) and PF-00835231, where the C-terminal residue reacts covalently with the nucleophilic cysteine have been developed^38,42,46–49^ (Extended Data Fig. 5a,b). X^Q^, which is present in these inhibitors, is an analogue of the substrate Q/P1 residue wherein the side chain δ-nitrogen is covalently linked to the γ-carbon (Extended Data Fig. 5b, indicated by the blue line) to give a conformationally constrained (*S*)-γ-lactam, which is well accommodated in the S1 subsite, resulting in improved potency relative to Q in inhibitors^46,50^. GM4 and GM4H3Q contain a related cγAA ring but are non-covalently binding inhibitors, but notably still exhibit low-nanomolar IC_50_ values.

Recently, non-covalent small molecule inhibitors against M^pro^ have also been developed by means of virtual screening and optimization of compounds based on structure–activity relationships^51–54^. Small molecular inhibitors often have an advantage of better cell membrane permeability than macrocycles and thus are able to inhibit M^pro^ in cells. In fact, an impressive M^pro^ non-covalent inhibitor, which is a current clinical candidate, S-217622, exhibited antiviral activity in cells (half-maximal effective concentration (EC_50_) = 0.37 μM, in Extended Data Fig. 5c)^51^. Another example of a non-covalent inhibitor, compound 23, is reported to have potent in vitro inhibitory activity (IC_50_ = 20 nM), but exhibited cellular cytotoxicity (half-maximal cytotoxic concentration (CC_50_) in Vero E6 cells = 1.15 μM)^52^. This contrasts with S-217622, for which cell cytotoxicity was not reported, presumably because of substantial medicinal chemistry efforts to optimize in vivo properties, including from a toxicity perspective. However, classical small molecule drugs often suffer from insufficient target specificity and unwanted cell cytotoxicity. By contrast, macrocyclic peptide inhibitors generally have remarkably high target specificities, leading to low cytotoxicity; however, at least for intracellular targets, relatively low membrane permeability can be an issue for peptide macrocycles. Our GM4 and related macrocycles show no cytotoxicity at concentrations as high as 100 µM, but as yet do not manifest obvious antiviral activity in cells, likely due to their poor membrane permeability. Johansen-Leete et al. have reported an M^pro^-inhibiting thioether-macrocyclic peptide using the same technology as that used by us, except without elongation reprogramming (compound 1 in Extended Data Fig. 5a)^43^. The originally discovered peptide showed a potent in vitro efficacy (*K*_i_ = 14 nM) but no antiviral activity. However, an increase in cellular uptake, by attaching a cell-penetrating peptide (CPP) at its C-terminal region, resulted in modest but observable antiviral activity. It is known that the addition of CPP to a macrocycle can not only increase cell permeability but also increase cytotoxicity depending on the CPP sequence and cell types used for the study^55,56^. Since we have witnessed improvements in in vivo potency by reengineering the macrocyclic peptide backbone structures, such as by *N*-methylation, in our previous works^57,58^, our ultimate goal is to generate a non-CPP-modified macrocycle with better cell membrane permeability and potent antiviral activity. In the case of the GM4 peptides, our current efforts are directed at reducing molecular weight while maintaining the critical yFHγ^1^ binding motif^59^.

Most importantly, our strategy for the construction of a cγAA-containing macrocyclic peptide library and its application for the RaPID system has demonstrated that unprecedented protease inhibitors containing cγAAs can be generated. It is striking that crystallography reveals that the γ^1^ residue of GM4 sits deeply in the M^pro^ active site, and that the compactness of γ^1^ plays a critical role in organizing a unique turn conformation, resulting in resistance to proteolytic cleavage of GM4. The general ability of cγAAs in the preorganization of turn conformations thus appears to be advantageous in obtaining potent, compact and stable macrocycles with drug-like properties.

## Methods

### Preparation of flexizymes and tRNAs

Flexizymes (dFx and eFx) and tRNAs for charging cγAAs, cβAAs and d-amino acids were transcribed in vitro using the T7 RNA polymerase from the corresponding template DNAs and prepared by extension and PCR (Supplementary Table 3 for primer sequences). PCR products were purified by phenol/chloroform extraction and ethanol precipitation. The transcription reaction was then carried out using the following mixture: 40 mM Tris-HCl buffer (pH 8.0), 22.5 mM MgCl_2_, 10 mM dithiothreitol, 1 mM spermidine, 0.01% Triton X-100, 3.75 mM NTP mix, 0.04 U μl^–1^ RNasin RNase inhibitor (Promega, N2615) and 120 nM T7 RNA polymerase (37 °C, 16 h). For transcription of tRNAs, 5 mM GMP was added to the above solution to introduce a monophosphate at the 5′ end of tRNAs. The resulting RNA transcripts were then treated with RQ1 DNase (Promega, M6101) at 37 °C for 30 min and purified by 12% (flexizymes) or 8% (tRNAs) denaturing polyacrylamide gel electrophoresis (PAGE) containing 6 M urea.

### Preparation of aminoacyl-tRNAs

Preparation of aminoacyl-tRNA employed the flexizyme method^25,33,29^. The cγAAs, cβAAs and d-cysteine (c) were pre-activated as their 3,5-dinitrobenzyl esters; *N*-chloroacetyl-d-tyrosine (^ClAc^y) was activated as its cyanomethyl ester. The activated amino acids were charged onto the respective tRNAs using flexizymes (dFx for 3,5-dinitrobenzyl ester or eFx for cyanomethyl ester). Aminoacylation was carried out at 4 °C for 16 h for cγAA and cβAA, 6 h for c or 2 h for ^ClAc^y in the following mixture: 600 mM MgCl_2_, 20% DMSO solvent, 25 μM dFx or eFx, 25 μM tRNA and 5 mM activated amino acid. The reaction pH was adjusted by bicine-KOH (pH 8.7) for cγAAs and cβAAs, or HEPES-KOH buffer (pH 7.5) for c and ^ClAc^y. The reaction was stopped by addition of ×4 volume of 0.3 M sodium acetate (pH 5.2) and ×10 volume of ethanol. The resulting aminoacyl-tRNAs were purified by ethanol precipitation.

### Translation of peptides

Ribosomal synthesis of the model peptides and the peptide library was carried out using the modified FIT system (37 °C, 40 min)^32,34^, which employs 3 μM initiation factor 2 (IF2), 20 μM EF-Tu, 5 μM EF-P and 0.1 μM elongation factor G (EF-G), and the following components: 50 mM HEPES-KOH (pH 7.6), 100 mM KOAc, 12.3 mM Mg(OAc)_2_, 2 mM ATP, 2 mM GTP, 1 mM CTP, 1 mM UTP, 20 mM creatine phosphate, 2 mM spermidine, 1 mM dithiothreitol, 1.5 mg ml^–1^ *E. coli* total tRNA, 1.2 μM *E. coli* ribosome, 2.7 μM IF1, 1.5 μM IF3, 0.25 μM release factor 2 (RF2), 0.17 μM RF3, 0.5 μM ribosome recycling factor (RRF), 4 μg ml^–1^ creatine kinase, 0.1 μM T7 RNA polymerase, 3 μg ml^–1^ myokinase, 0.1 μM inorganic pyrophosphatase, 0.1 μM nucleotide diphosphate kinase, l-α-amino acids (500 μM each G/H/N/P/R/T/Y, 250 μM each D/F/I/L/S/K and 125 μM A), 0.73 μM AlaRS, 0.03 μM ArgRS, 0.38 μM AsnRS, 0.13 μM AspRS, 0.09 μM GlyRS, 0.02 μM HisRS, 0.4 μM IleRS, 0.04 μM LeuRS, 0.11 μM LysRS, 0.68 μM PheRS, 0.16 μM ProRS, 0.04 μM SerRS, 0.09 μM ThrRS, 0.02 μM TyrRS, 50 μM γ^1^-tRNA^Pro1E2^_CUC_, 100 μM γ^2^-tRNA^Pro1E2^_CAC_, 20 μM β^1^-tRNA^Pro1E2^_GAC_, 20 μM β^2^-tRNA^GluE2^_GCA_, 20 μM ^ClAc^y-tRNA^fMet^_CAU_ and 20 μM c-tRNA^Pro1E2^_CAU_. For translation of model peptides, a 0.04 μM DNA template was added to the above solution for transcription/translation coupled reactions. The DNA templates were prepared by extension and PCR (Supplementary Table 4 for the primer sequences). For the translation of the peptide library, a 1.5 μM mRNA library conjugated to puromycin linker was used.

### MALDI-TOF MS of translated model peptides

The model peptides with a C-terminal FLAG-tag (DYKDDDDK) were translated and diluted with an equal volume of ×2 TBS buffer (100 mM Tris-HCl (pH 7.6), 300 mM NaCl), and then incubated with 10 μl of ANTI-FLAG M2 affinity gel (Sigma, A2220) at 25 °C for 30 min. The gel beads were washed with 50 μl TBS buffer (50 mM Tris-HCl (pH 7.6), 150 mM NaCl); the peptides were eluted from the beads by adding 20 μl of 0.2% (v/v) trifluoroacetic acid (TFA). The peptides were desalted with SPE C-tip (Nikkyo Technos) and eluted with 1.2 μl of 80% (v/v) acetonitrile and 0.5% (v/v) acetic acid solution containing 50%-saturated α-cyano-4-hydroxycinnamic acid. MALDI-TOF MS was carried out using an ultrafleXtreme instrument (Bruker Daltonics) in reflector/positive mode. Peptide calibration standard II (Bruker Daltonics, 8222570) was used for the external mass calibration.

### Production of recombinant SARS-CoV-2 M^pro^

M^pro^ was prepared as reported, and assays with it were performed exclusively using freshly purified recombinant M^pro^ solution^39,61^. Refrozen M^pro^ samples exhibited reduced activity and were not used.

### RaPID selection of peptides against SARS-CoV-2 M^pro^

The random mRNA library was ligated with a puromycin linker at the 3′ end, and then added to the above described translation mixture (Extended Data Fig. 1a, step 1). The peptide library was translated at 37 °C for 40 min in 150 μl (for the first round of selection) or 10 μl (for the second to fourth rounds) of the FIT system and incubated at 25 °C for 5 min to conjugate the translated peptide with the corresponding mRNA–puromycin (step 2). A ×0.04 volume of 500 mM ethylenediaminetetraacetic acid (EDTA; pH 8.0) was then added and incubated (37 °C, 10 min) to dissociate ribosomes from the mRNA–peptide conjugates. Reverse transcription (42 °C, 30 min) used the NNUGWGAUG.R38 primer (5′-TTTCCGCCCCCCGTCCTAACCTCCGCTACCTCCACTCA-3′) and M-MLV reverse transcriptase lacking RNase H activity (Promega, M3682; step 3). The resulting cDNA/mRNA/peptide conjugates were subjected to naked Dynabead streptavidin (Thermo Fisher, DB11206) treatment (4 °C, 15 min) three times to remove bead-binding peptides; the supernatant was then applied to M^pro^-immobilized Dynabeads (4 °C, 15 min; step 4). The beads were washed with 100 μl ice-cold TBS-T buffer (50 mM Tris-HCl (pH 7.6), 150 mM NaCl, 0.05% (v/v) Tween 20) three times. Note that the removal of bead-binding peptides was not performed for the first selection round. Then, 100 μl of ×1 PCR buffer (10 mM Tris-HCl (pH 9.0), 50 mM KCl, 0.1% (v/v) Triton X-100, 0.25 mM dNTP, 2.5 mM MgCl_2_, 0.25 μM T7.F53 primer (5′-GGCGTAATACGACTCACTATAGGGTTGAACTTTAAGTAGGAGATATATCCATG-3′) and 0.25 μM NNUGWGAUG.R38 primer) was added to the beads; the cDNAs were eluted at 95 °C for 5 min and PCR amplified to make a cDNA library (step 5). To estimate the recovery rate of cDNA, 1 μl of the elute was mixed with 19 μl of ×1 PCR buffer containing SYBR Green I (Lonza, 50513) and *Taq* DNA polymerase; amounts of cDNA were quantified by real-time PCR.

### Solid-phase peptide synthesis

Macrocyclic peptides were synthesized by standard Fmoc solid-phase peptide synthesis using a Syro I automated peptide synthesizer (Biotage). Fmoc-protected amino acids and coupling reagents were from Merck, Watanabe Chemical Industries or Enamine. NovaPEG Rink Amide Resin (54 mg, 25 μmol) was incubated with *N*,*N*-dimethylformamide (DMF; room temperature, 1 h). Each Fmoc-protected amino acid was coupled at 30 °C for 40 min on the resin in a DMF solution containing 0.2 M Fmoc-protected amino acid (6 equiv.), 0.2 M 2-(1*H*-benzotriazole-1-yl)-1,1,3,3-tetramethyluronium hexafluorophosphate (5 equiv.), 0.2 M 1-hydroxybenzotriazole (5 equiv.) and 0.1 M *N*,*N*-diisopropylethylamine (12 equiv.). After washing the resin five times with 600 μl DMF, the Fmoc group was deprotected with 600 μl of 40% (v/v) piperidine in DMF at 30 °C for 12 min. Coupling of the Fmoc-protected amino acid and Fmoc deprotection were repeated accordingly. After automated peptide synthesis, 0.2 M chloroacetyl *N*-hydroxysuccinimide ester (8 equiv.) in *N*-methylpyrrolidone was added to the resin; the mixture was incubated at room temperature for 1 h with rotation. After washing the resin subsequently with DMF five times and with dichloromethane five times, the resin-bound peptides were treated with 2 ml of a solution of 92.5% (v/v) TFA, 2.5% (v/v) water, 2.5% triisopropylsilane and 2.5% 3,6-dioxa-1,8-octanedithiol at room temperature for 3 h with rotation to deprotect and cleave off from the resin. The resulting linear peptides were precipitated with diethyl ether and dissolved in 10 ml of 80% (v/v) DMSO and 0.1% TFA in water. Following the addition of 200 μl of 0.5 M tris(2-carboxyethyl) phosphine and triethylamine to adjust the pH to 8, the peptide mixture was incubated with rotation at room temperature for 10 h to form a thioether bond between the N-terminal chloroacetamide and thiol group of the downstream cysteine. Macrocyclization of the peptides was confirmed by MALDI-TOF MS; the crude peptides were purified by reverse-phase high-performance LC (Shimadzu). The purities of the peptides were evaluated by UPLC (Shimadzu) using a reverse-phase column (ACQUITY UPLC BEH C18, 1.7 μm, 2.1 × 150 mm; Waters) with a linear gradient from 10% buffer B to 70% buffer B. Buffer A was water with 0.1% (v/v) TFA; buffer B was acetonitrile with 0.1% (v/v) TFA.

### Binding kinetics analysis of peptides by SPR

The binding affinities of selected peptides and M^pro^ were analysed by SPR using a Biacore T200 instrument (Cytiva) at 25 °C. The composition of the running buffer was 10 mM Tris-HCl (pH 8.0), 150 mM NaCl, 0.05% (v/v) Tween 20 and 0.1% (v/v) DMSO. Biotin-tagged M^pro^ was immobilized on a Biacore sensor chip CAP (Cytiva) to a surface density of 1,500–2,000 response units following the immobilization protocols of the Biotin CAPture Kit (Cytiva). The kinetic constants were determined by a single-cycle kinetics method by the injection of five different concentrations (twofold dilution series) of each peptide at a flow rate of 30 μl min^–1^. Binding sensorgrams were fitted to the standard 1:1 interaction model and analysed using Biacore evaluation software.

### Solid-phase extraction coupled to MS inhibition assays

Inhibition of M^pro^ was measured by solid-phase extraction purification coupled to MS analysis using a RapidFire 365 high-throughput sampling robot (Agilent) connected to an iFunnel Agilent 6550 accurate mass quadrupole TOF spectrometer as reported^39^. In brief, the cyclic peptides were dispensed in an 11-point, threefold dilution series across 384-well plates using an acoustic Echo Dispenser machine (LabCyte). Formic acid and DMSO were used as positive and negative controls, respectively. The assay was adapted for a reaction volume of 20 μl with a final condition of 75 nM M^pro^ and 4 µM 37-mer substrate (ALNDFSNSGSDVLYQPPQTSITSAVLQ/SGFRKMAFPS-NH_2_). Reactions were incubated (15 min) and then quenched by the addition of 10% (v/v) aqueous formic acid (5 μl per well). C-terminal product peptide-derived SGFRKMAF-NH_2_ (10 μl per well) was employed as the internal standard; data were extracted and processed as reported.

### Serum stability assays

A synthetic macrocyclic peptide (10 μM) and an internal standard peptide (10 μM) (Supplementary Fig. 6a for its structure) were mixed and incubated in human serum (Cosmo Bio, 12181201) at 37 °C for up to 100 h. We have previously shown that the standard peptide is peptidase resistant, with no degradation occurring under these experimental conditions^25^. At each time point (0, 0.5, 1, 2, 4, 8, 24, 50 and 100 h), 4 μl of the mixture was removed and quenched by adding 12 μl methanol. Following centrifugation (15,000*g*, 25 °C, 3 min), 10 μl of the supernatant was mixed with 40 μl of 1% (v/v) TFA. Following centrifugtion (15,000*g*, 25 °C, 3 min), the supernatant was collected for LC/MS analysis, which used a reverse-phase column (ACQUITY UPLC BEH C18, 1.7 μm, 2.1 × 150 mm; Waters) and a Xevo G2-XS QTof system (Waters) with a linear gradient from 1% B to 60% B. Buffer A was water with 0.1% (v/v) formic acid; buffer B was acetonitrile with 0.1% (v/v) formic acid. The percentages of remaining peptides were determined by the peak area integration of the chromatograms.

### Crystallization, data collection and structure solution

M^pro^ was thawed and diluted to 6 mg ml^–1^ using 20 mM HEPES (pH 7.5) and 50 mM NaCl. GM4 was diluted into the protein solution to a final concentration of 10 mM, which was incubated for two hours at room temperature prior to dispensing plates. The drop composition was 0.15 μl protein ligand solution, 0.3 μl 11% (v/v) polyethylene glycol (PEG) 4K, 0.1 M 2-(*N*-morpholino)ethanesulfonic acid (MES) pH 6.5 and 0.05 μl M^pro^ crystal seed stock. The M^pro^ crystal seed stock was prepared by crushing M^pro^ crystals with a pipette tip; suspending them in 30% PEG 4K, 5% (v/v) DMSO and 0.1 M MES pH 6.5; and vortexing for 60 s with ~10 glass beads (1.0 mm diameter, BioSpec Products). The reservoir solution was 11% (v/v) PEG 4K, 5% (v/v) DMSO and 0.1 M MES pH 6.5. Crystals were grown by the sitting drop vapour diffusion method (20 °C) and appeared within 24 h, reaching full size within 36 h; they were harvested after one week.

#### Data collection and structure determination

All diffraction data were collected at 100 K with a wavelength of 0.9762 Å on beamline I03 at the Diamond Light Source. Data were processed using Dials^62^ via Xia2^63^ and Aimless^64^ within CCP4i2 (ref. ^65^). The datasets were phased using Molrep^66^ using the M^pro^ apo structure (Protein Data Bank 6YB7). Ligand restraints were generated using AceDRG^67^. Crystal structures were manually rebuilt in Coot and refined using Refmac^68^ and PDBredo^69^.

### Reporting summary

Further information on research design is available in the Nature Portfolio Reporting Summary linked to this article.

## Online content

Any methods, additional references, Nature Portfolio reporting summaries, source data, extended data, supplementary information, acknowledgements, peer review information; details of author contributions and competing interests; and statements of data and code availability are available at 10.1038/s41557-023-01205-1.

## Supplementary information

Supplementary Information Supplementary Figs. 1–6.

Reporting Summary

Supplementary Table 1, 2, 3 and 4 Top 100 peptide sequences of M^pro^ binders after the fourth round of selection.

## Data Availability

Coordinates and structure factors have been deposited in the Protein Data Bank (PDB) under accession code 7Z4S. Other data supporting this study are available in the Supplementary Information. Source data are provided with this paper.

## Source data

Source Data Fig. 1 Raw data of inhibitory activity assays and serum stability assays.
