# Supplementary material for: In vitro selection of macrocyclic peptide inhibitors containing cyclic γ2,4-amino acids targeting the SARS-CoV-2 main protease
Source: Nat Chem. 2023 May 22;15(7):998–1005. doi: 10.1038/s41557-023-01205-1 (PMC10322702; doi:10.1038/s41557-023-01205-1)
Supplement: Supplementary file 1 — Supplementary Figs. 1–6. [file 41557_2023_1205_MOESM1_ESM.pdf]

nature chemistry

Article https://doi.org/10.1038/s41557-023-01205-1

In vitro selection of macrocyclic peptide inhibitors containing cyclic $\gamma^{2,4}$-amino acids targeting the SARS-CoV-2 main protease

In the format provided by the authors and unedited

Supplementary Information for:

In vitro selection of macrocyclic peptide inhibitors containing cyclic $\gamma^{2,4}$-amino acids targeting the SARS-CoV-2 main protease

Takashi Miura[1], Tika R. Malla[2], C. David Owen[3,4], Anthony Tumber[2], Lennart Brewitz[2], Michael A. McDonough[2], Eidarus Salah[2], Naohiro Terasaka[1], Takayuki Katoh[1], Petra Lukacik[3,4], Claire Strain-Damerell[3,4], Halina Mikolajek[3,4], Martin A. Walsh[3,4], Akane Kawamura[2,5], Christopher J. Schofield[2], and Hiroaki Suga[1]*

[1]Department of Chemistry, Graduate School of Science, The University of Tokyo, 7-3-1 Hongo, Bunkyo-ku, Tokyo 113-0033, Japan
[2]Department of Chemistry and the Ineos Oxford Institute for Antimicrobial Research, Chemistry Research Laboratory, University of Oxford, 12 Mansfield Road, OX1 3TA, Oxford, United Kingdom
[3]Diamond Light Source, Harwell Science & Innovation Campus, Didcot, Oxfordshire OX11 0DE, United Kingdom
[4]Research Complex at Harwell, Harwell Science & Innovation Campus, Didcot OX11 0FA, United Kingdom
[5]Chemistry – School of Natural and Environmental Sciences, Newcastle University, Newcastle upon Tyne, NE1 7RU, United Kingdom.

*To whom correspondence should be addressed.
E-mail: hsuga@chem.s.u-tokyo.ac.jp (H.S.)

Table of Contents

Title	Page
Supplementary Fig. 1: Secondary structures of tRNAs used for incorporation of cγAAs, cβAAs, and D-amino acids.	3
Supplementary Fig. 2: Ribosomal synthesis of model macrocyclic peptides containing cγAA or cβAA.	4
Supplementary Fig. 3: Purities and identities of peptides synthesized by SPPS.	5
Supplementary Fig. 4: Chemical structures of selected peptides by RaPID including the most potent variant GM4H3Q.	7
Supplementary Fig. 5: Binding kinetics of macrocyclic peptides against M^{pro}.	9
Supplementary Fig. 6: Serum stability assay of macrocyclic peptides.	10

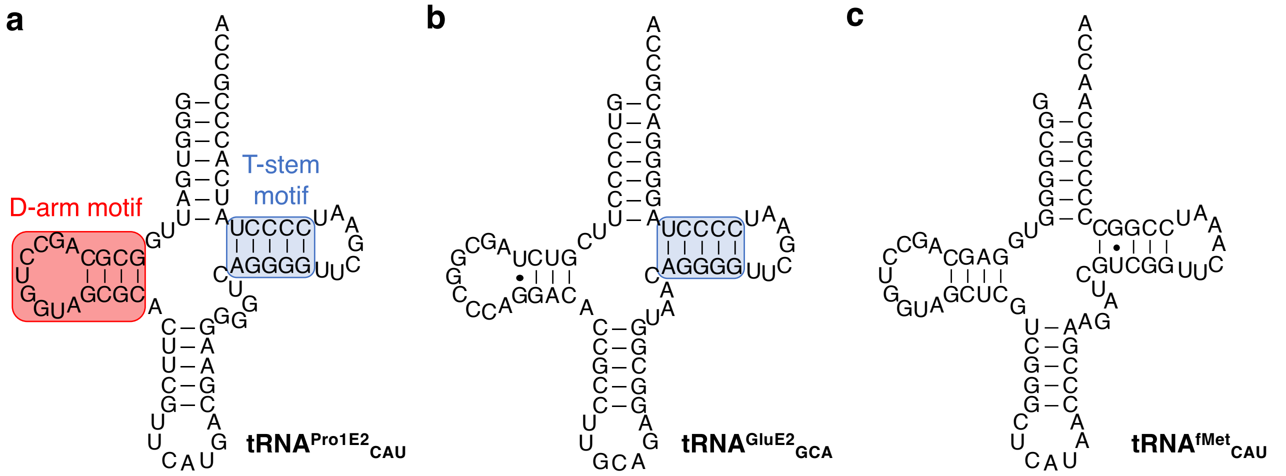

Supplementary Fig. 1: Secondary structures of tRNAs used for incorporation of cγAAs, cβAAs, and D-amino acids. **a**, $\text{tRNA}^{\text{Pro1E2}}_{\text{CAU}}$ used for decoding AUG codons. For decoding other codons, the sequence of anticodon loop was changed accordingly (see Supplementary Table 3 for the sequences). The D-arm motif for EF-P binding consists of a 9-nt D-loop closed by a stable 4-bp stem (highlighted in red). The T-stem motif for EF-Tu binding is highlighted in blue. **b**, $\text{tRNA}^{\text{GluE2}}_{\text{GCA}}$ used for decoding UGC codons. The T-stem motif for EF-Tu binding highlighted in blue is identical to that of $\text{tRNA}^{\text{Pro1E2}}$. **c**, $\text{tRNA}^{\text{fMet}}_{\text{CAU}}$ used for incorporation of ^{ClAc}y at the initiator AUG codon.

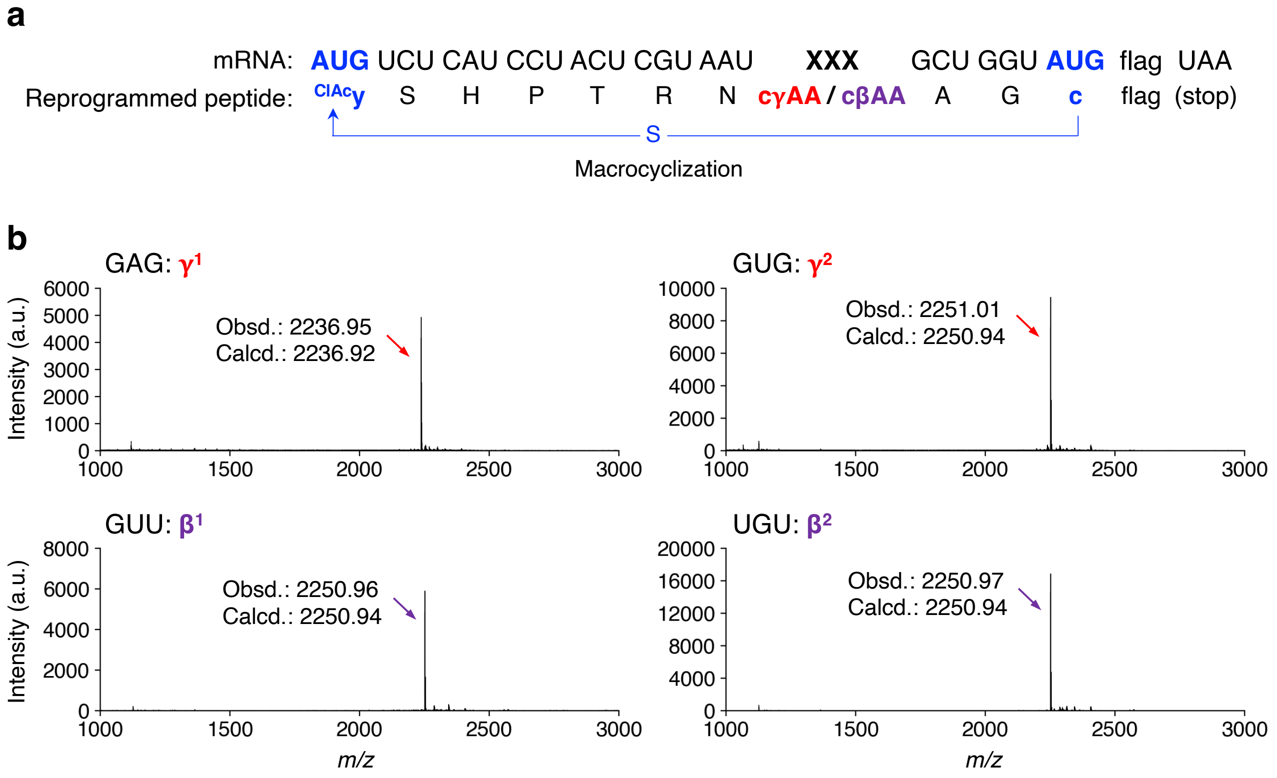

Supplementary Fig. 2: Ribosomal synthesis of model macrocyclic peptides containing cγAA or cβAA. a, Sequence of template mRNA for a model peptide. XXX indicates the codon used for incorporation of cγAA or cβAA. **b**, MALDI-TOF mass spectra of model peptides. Codons used for incorporation of cγAA or cβAA are indicated. Calcd. and Obsd. indicate calculated and observed $[M+H]^+$ values, respectively.

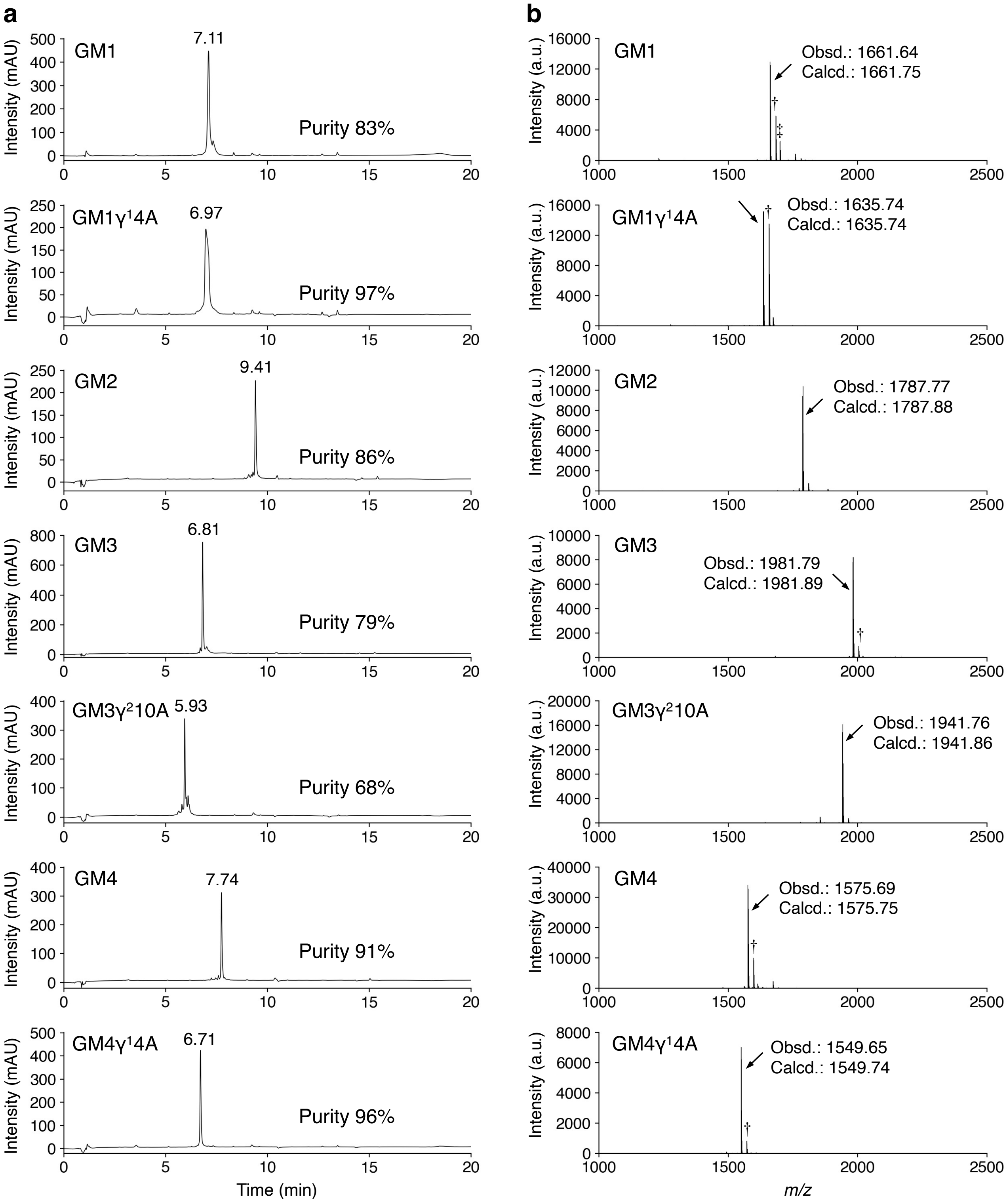

Supplementary Fig. 3: Purities and identities of peptides synthesized by SPPS. a,**c**, UV (λ = 280 nm) chromatograms of peptides by UPLC. Purity was determined by calculating the ratio of peak area of desired peptide relative to the combined areas of all peaks present. **b**,**d**, MALDI-TOF mass spectra of peptides. Calcd. and Obsd. indicate calculated and observed $[M+H]^+$ values, respectively. † and ‡ indicate sodium and potassium ion adducts, respectively.

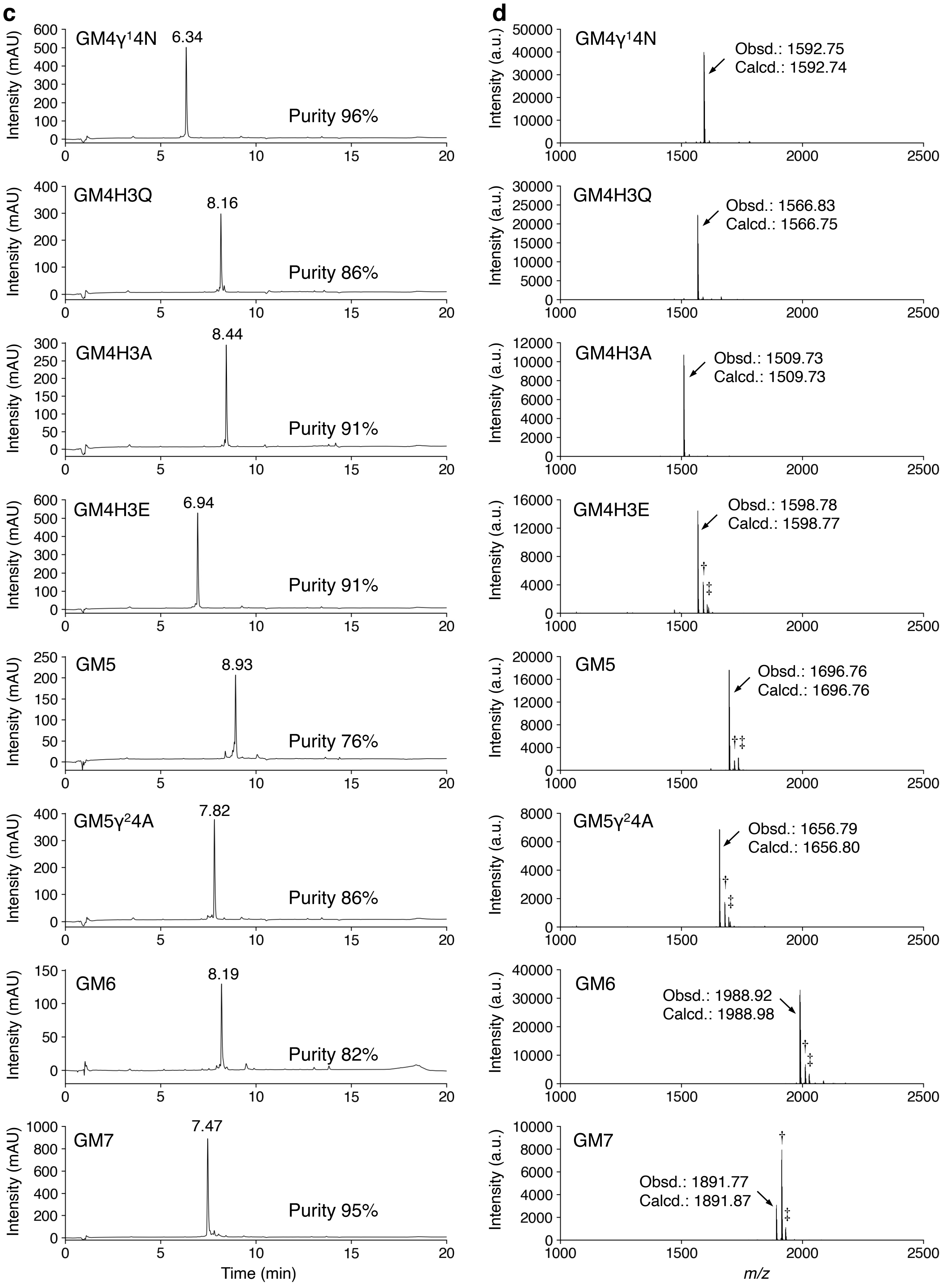

Supplementary Fig. 3, continued.

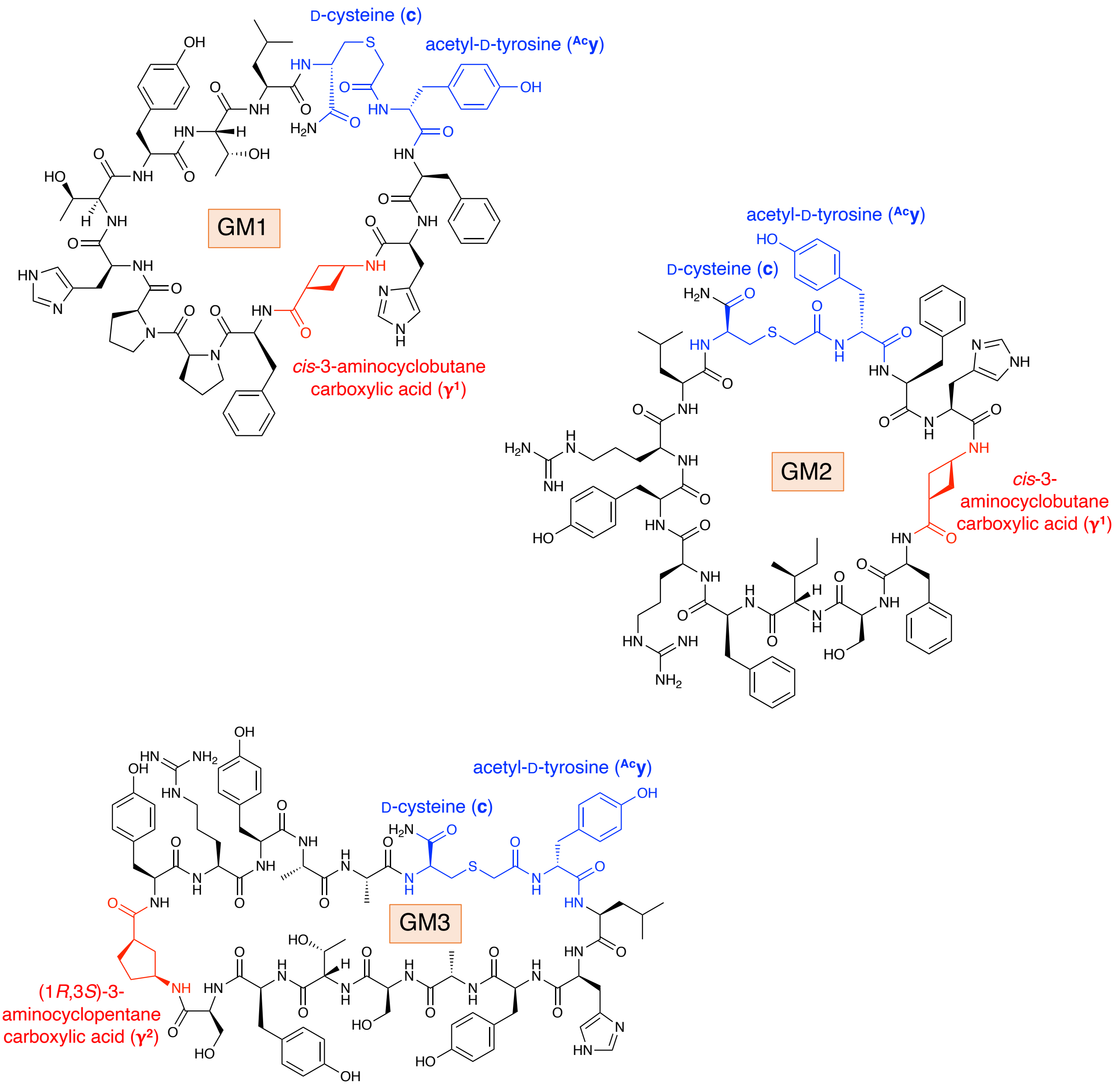

Supplementary Fig. 4: Chemical structures of selected peptides by RaPID including the most potent variant GM4H3Q. See Fig. 3b for GM4.

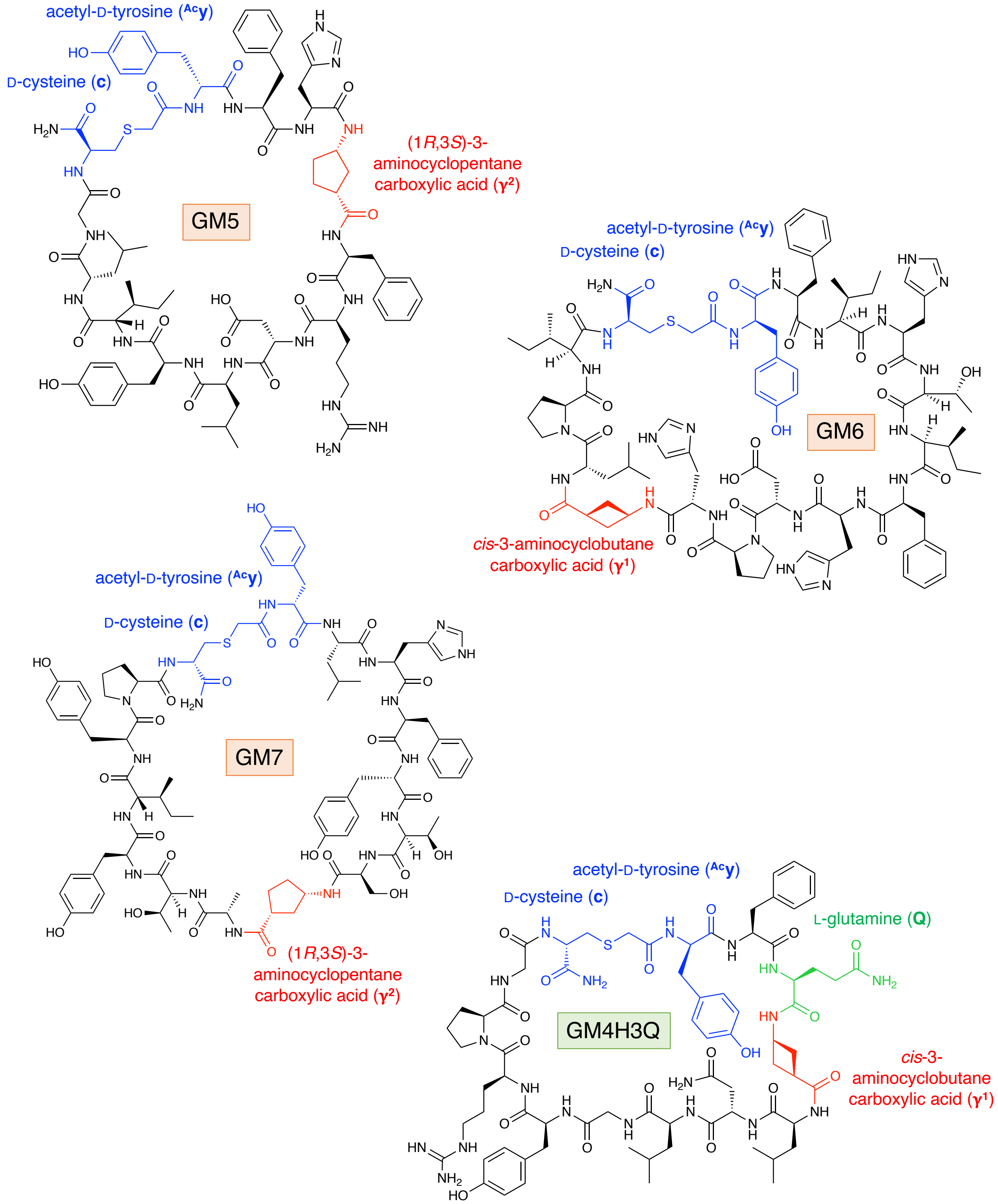

Supplementary Fig. 4, continued.

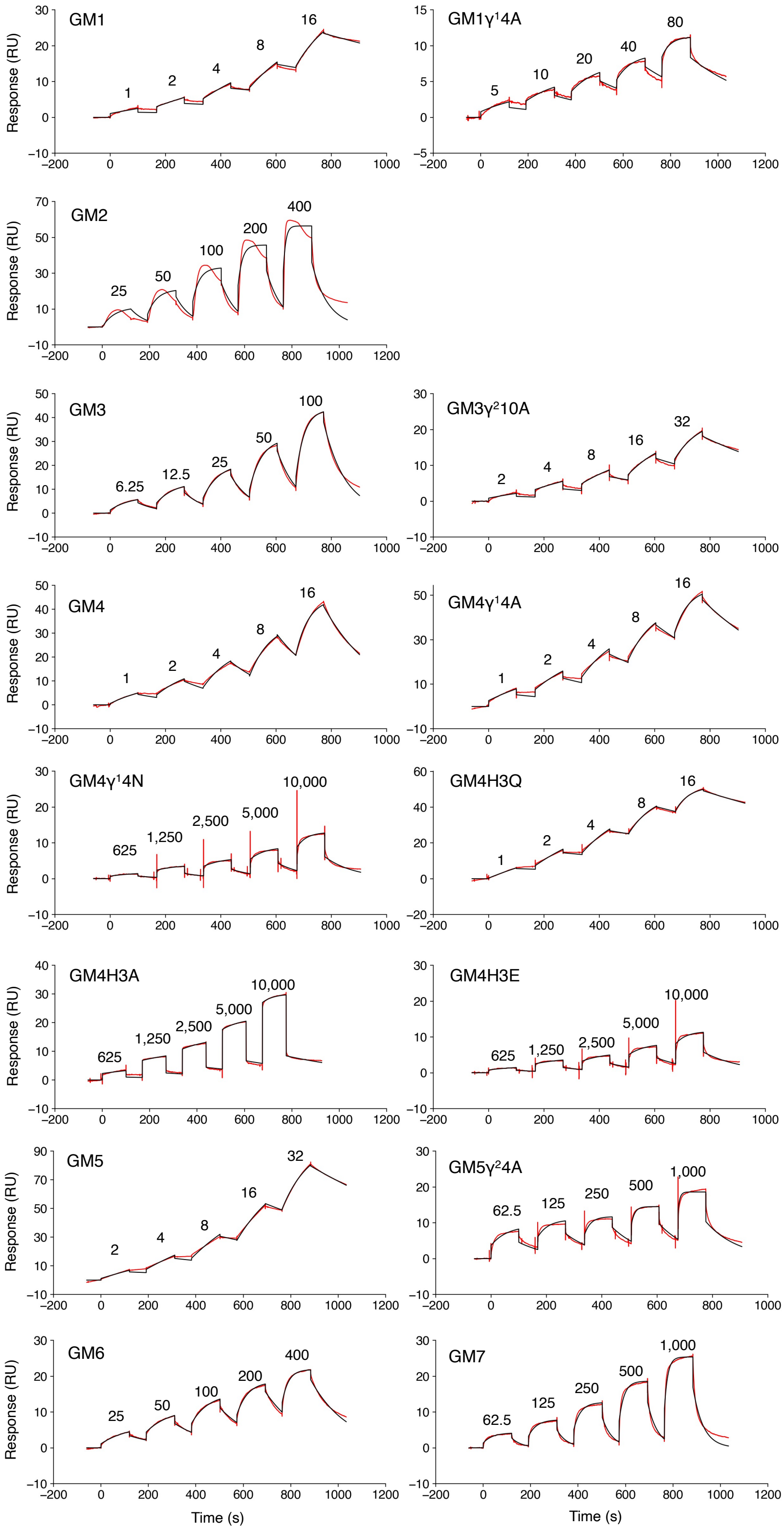

Supplementary Fig. 5: Binding kinetics of macrocyclic peptides against M^{pro}. SPR sensorgrams of selected peptides and variants. The sequences and kinetic values are shown in Table 1. Five different concentrations (nM), indicated by the numbers above the sensorgrams, of each peptide were injected for measuring kinetic constants. Binding sensorgrams were fitted using the standard 1:1 binding model. Red and black lines indicate raw sensorgrams and fitted curves, respectively.

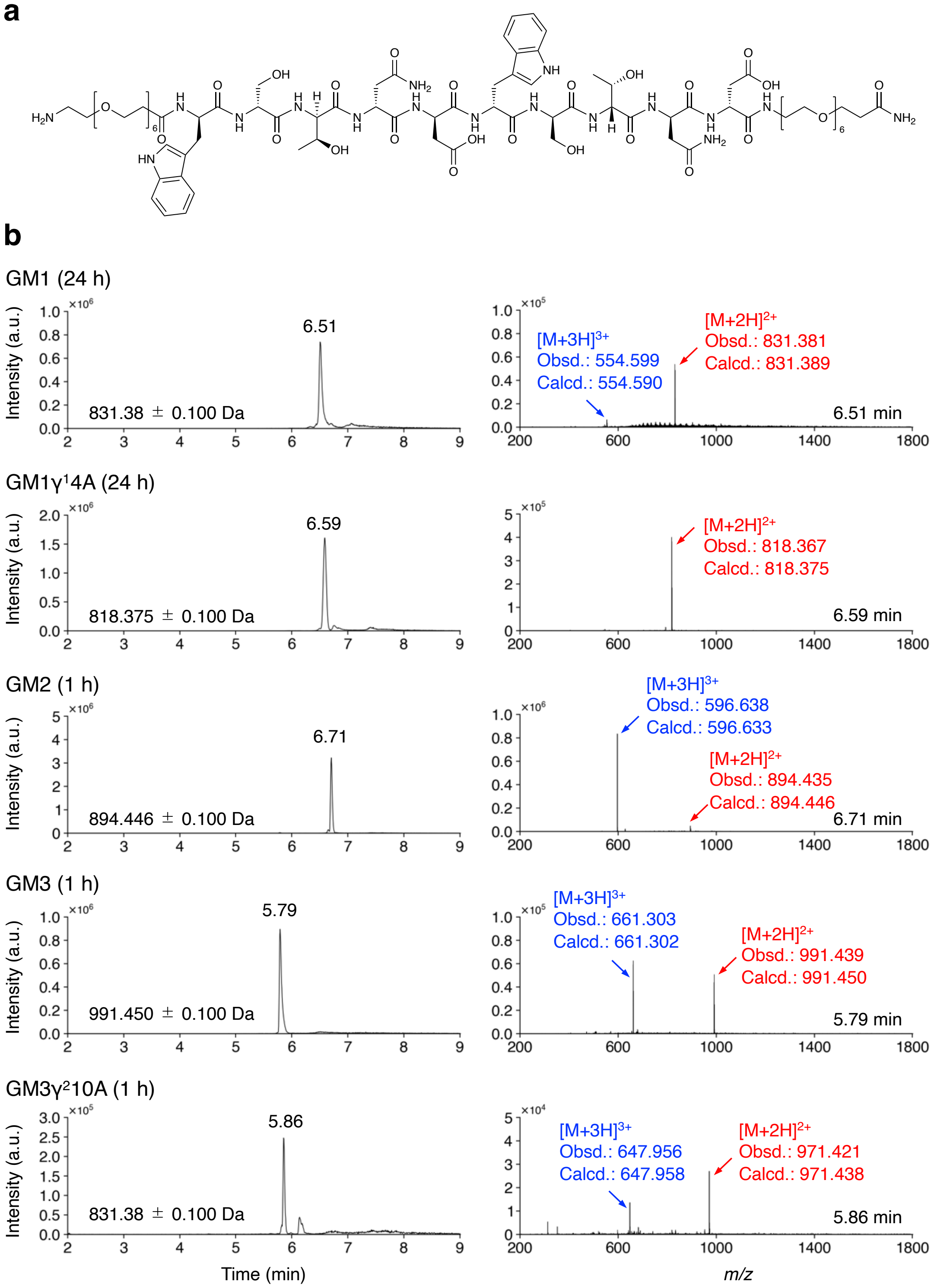

Supplementary Fig. 6: Serum stability assay of macrocyclic peptides. a, Structure of the internal standard peptide used in serum stability assays. **b**,**c**, Chromatogram and mass spectra of reaction mixture after incubation for 24 h (GM1, GM1γ[1]4A, GM4, GM4H3Q, GM5, and GM5γ[2]4A) or 1 h (GM2, GM3, GM3γ[2]10A, and GM4γ[1]4A). Red and blue arrows indicate $[M+2H]^{2+}$ and $[M+3H]^{3+}$ ions, respectively. Calcd. and Obsd. stand for calculated and observed *m/z* values of peptides. **d**,**e**, Chromatogram and mass spectra of fragments GM4-f1−6 and GM4γ[1]4A-f1−3, respectively. Red and black arrows indicate the $[M+2H]^{2+}$ and $[M+H]^{+}$ ions, respectively.

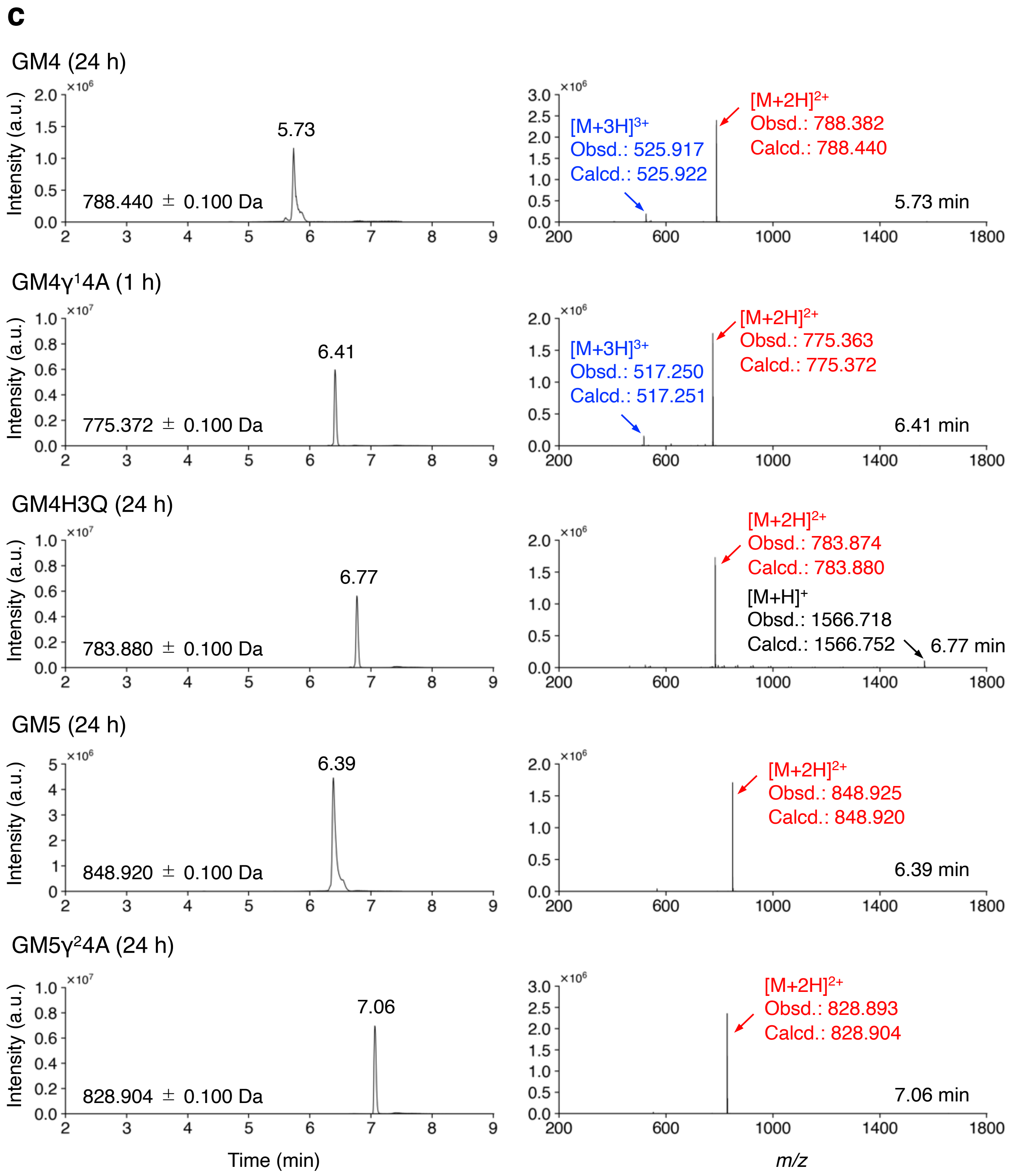

Supplementary Fig. 6, continued.

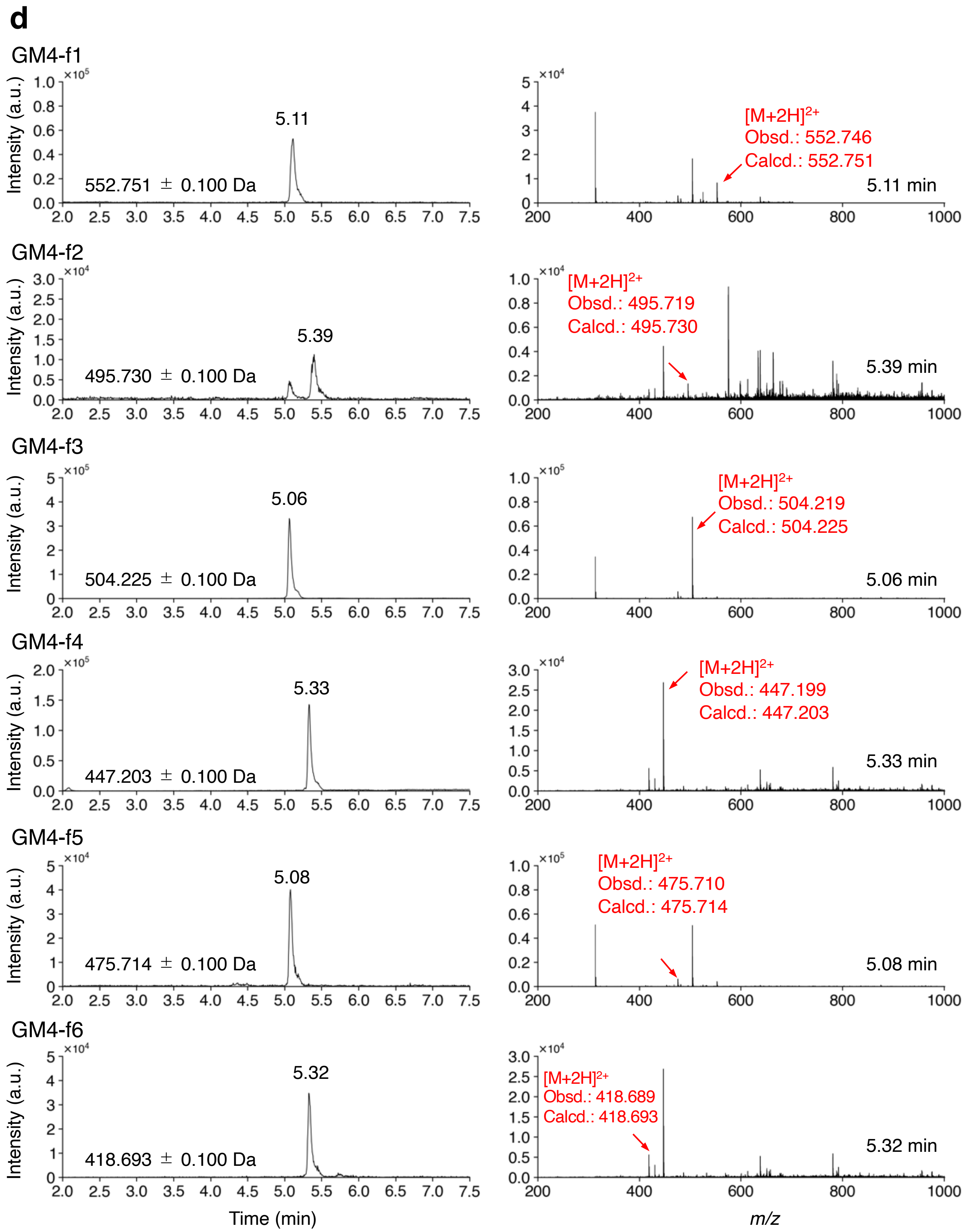

Supplementary Fig. 6, continued.

e

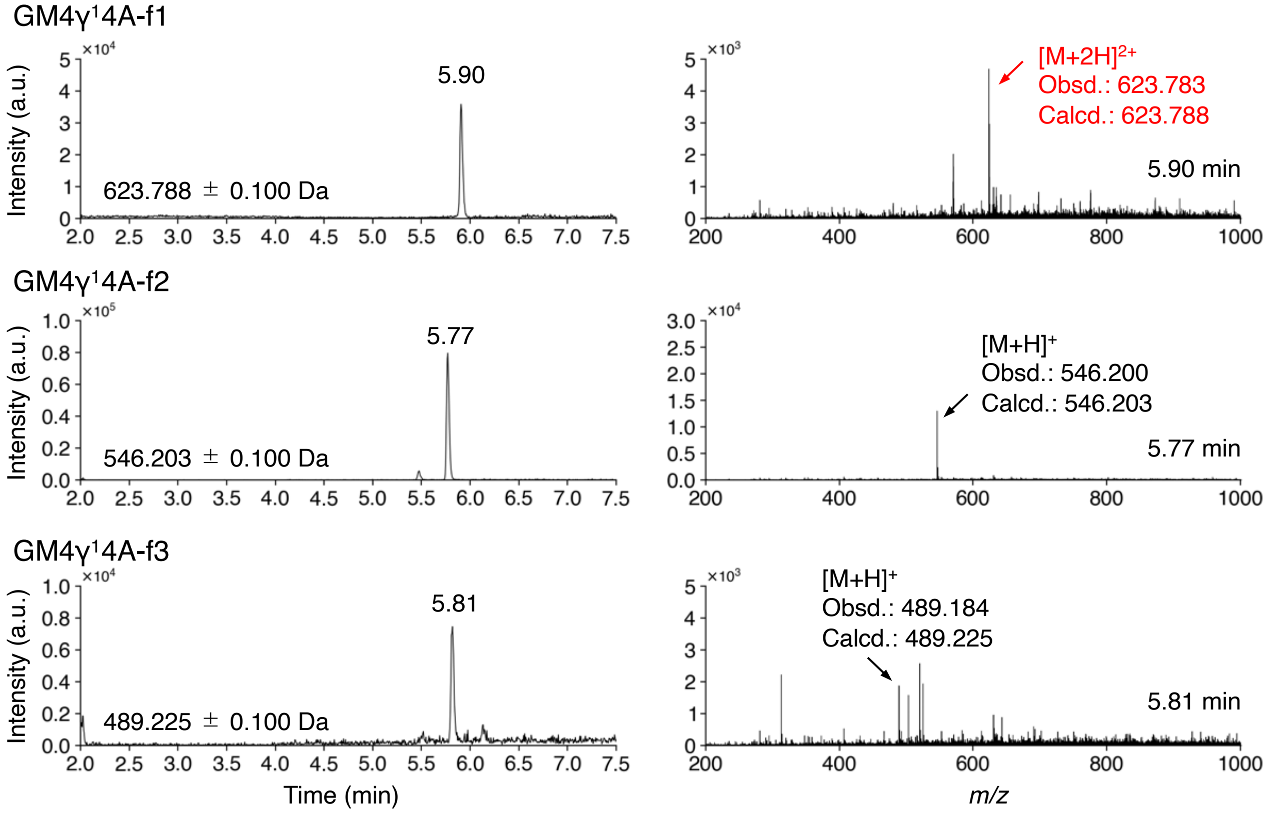

Supplementary Fig. 6, continued.
