# Supplementary material for: In vitro selection of macrocyclic peptide inhibitors containing cyclic γ2,4-amino acids targeting the SARS-CoV-2 main protease
Source: Nat Chem. 2023 May 22;15(7):998–1005. doi: 10.1038/s41557-023-01205-1 (PMC10322702; doi:10.1038/s41557-023-01205-1)
Supplement: Supplementary file 2 — Reporting Summary [file 41557_2023_1205_MOESM2_ESM.pdf]

nature portfolio

Corresponding author(s): Hiroaki Suga

Last updated by author(s): Jan 30, 2023

Reporting Summary

Nature Portfolio wishes to improve the reproducibility of the work that we publish. This form provides structure for consistency and transparency in reporting. For further information on Nature Portfolio policies, see our Editorial Policies and the Editorial Policy Checklist.

Statistics

For all statistical analyses, confirm that the following items are present in the figure legend, table legend, main text, or Methods section.

n/a	Confirmed	
☐	☒	The exact sample size (n) for each experimental group/condition, given as a discrete number and unit of measurement
☐	☒	A statement on whether measurements were taken from distinct samples or whether the same sample was measured repeatedly
☒	☐	The statistical test(s) used AND whether they are one- or two-sided<br>*Only common tests should be described solely by name; describe more complex techniques in the Methods section.*
☒	☐	A description of all covariates tested
☒	☐	A description of any assumptions or corrections, such as tests of normality and adjustment for multiple comparisons
☐	☒	A full description of the statistical parameters including central tendency (e.g. means) or other basic estimates (e.g. regression coefficient) AND variation (e.g. standard deviation) or associated estimates of uncertainty (e.g. confidence intervals)
☒	☐	For null hypothesis testing, the test statistic (e.g. F, t, r) with confidence intervals, effect sizes, degrees of freedom and P value noted<br>*Give P values as exact values whenever suitable.*
☒	☐	For Bayesian analysis, information on the choice of priors and Markov chain Monte Carlo settings
☒	☐	For hierarchical and complex designs, identification of the appropriate level for tests and full reporting of outcomes
☒	☐	Estimates of effect sizes (e.g. Cohen's d, Pearson's r), indicating how they were calculated

Our web collection on statistics for biologists contains articles on many of the points above.

Software and code

Policy information about availability of computer code

Data collection	MALDI-TOF MS data was collected on Bruker flexControl 3.4. Next generation sequencing data was collected on Illumina MiSeq Control Software 2.4.1.3. HPLC/UPLC data was collected on Shimadzu LabSolutions 5.99. SPR data was collected on Cytiva Biacore T200 Control Software 2.0.1. LC/MS data was collected on Waters MassLynx 4.2.
Data analysis	MALDI-TOF MS data was analyzed on Bruker flexAnalysis 3.4. HPLC/UPLC data was analyzed on Shimadzu LabSolutions 5.99. SPR data was analyzed on Cytiva Biacore T200 Evaluation Software 3.0. LC/MS data was analyzed on MassLynx 4.2. Kinetic data fitting was performed using GraphPad Prism 9.0.

For manuscripts utilizing custom algorithms or software that are central to the research but not yet described in published literature, software must be made available to editors and reviewers. We strongly encourage code deposition in a community repository (e.g. GitHub). See the Nature Portfolio guidelines for submitting code & software for further information.

Data

Policy information about availability of data

All manuscripts must include a data availability statement. This statement should provide the following information, where applicable:

- Accession codes, unique identifiers, or web links for publicly available datasets
- A description of any restrictions on data availability
- For clinical datasets or third party data, please ensure that the statement adheres to our policy

Coordinates and structure factors have been deposited in the Protein Data Bank, under accession code 7Z4S. Other data supporting this study are available in the Supplementary Information. Source data are provided with this paper. Other results are available from the corresponding author upon reasonable request.

Human research participants

Policy information about studies involving human research participants and Sex and Gender in Research.

Reporting on sex and gender	n/a
Population characteristics	n/a
Recruitment	n/a
Ethics oversight	n/a

Note that full information on the approval of the study protocol must also be provided in the manuscript.

Field-specific reporting

Please select the one below that is the best fit for your research. If you are not sure, read the appropriate sections before making your selection.

☒ Life sciences ☐ Behavioural & social sciences ☐ Ecological, evolutionary & environmental sciences

For a reference copy of the document with all sections, see nature.com/documents/nr-reporting-summary-flat.pdf

Life sciences study design

All studies must disclose on these points even when the disclosure is negative.

Sample size	Next generation sequencing (NGS) after selection was performed by selecting appropriate sequencing kit (MiSeq Reagent kit v3, 150-cycle) to read more than 10,000 sequences to confirm that the sequences had converged. Seven peptides with high read numbers (>200) were selected from the NGS results. Top 100 peptide sequences obtained by the NGS are listed in Supplementary Table 1.
Data exclusions	No data were excluded.
Replication	All assays were performed at least three times with the same results.
Randomization	There were no experiments for randomization. We selected peptide sequences containing γ-amino acids with high read numbers in the NGS results for subsequent assays.
Blinding	Not required for this study because there is no data selection in the experimental approach that could introduce bias.

Reporting for specific materials, systems and methods

We require information from authors about some types of materials, experimental systems and methods used in many studies. Here, indicate whether each material, system or method listed is relevant to your study. If you are not sure if a list item applies to your research, read the appropriate section before selecting a response.

Materials & experimental systems

n/a	Involved in the study
☒	☐ Antibodies
☒	☐ Eukaryotic cell lines
☒	☐ Palaeontology and archaeology
☒	☐ Animals and other organisms
☒	☐ Clinical data
☒	☐ Dual use research of concern

Methods

n/a	Involved in the study
☒	☐ ChIP-seq
☒	☐ Flow cytometry
☒	☐ MRI-based neuroimaging
